# Pan-Cancer Landscape of NEIL3 in Tumor Microenvironment: A Promising Predictor for Chemotherapy and Immunotherapy

**DOI:** 10.3390/cancers15010109

**Published:** 2022-12-24

**Authors:** Weixin Liao, Shaozhuo Huang, Lin Li, Jialiang Wang, Jing Li, Yongjian Chen, Lubiao Chen, Yifan Lian, Yuehua Huang

**Affiliations:** 1 Department of Infectious Diseases, The Third Affiliated Hospital of Sun Yat-sen University, Guangzhou 510630, China; 2 Guangdong Provincial Key Laboratory of Liver Disease Research, The Third Affiliated Hospital of Sun Yat-sen University, Guangzhou 510630, China; 3 Department of General Surgery, The Third Affiliated Hospital of Sun Yat-sen University, Guangzhou 510630, China; 4 Department of Medical Oncology, The Third Affiliated Hospital of Sun Yat-sen University, Guangzhou 510630, China

**Keywords:** NEIL3, pan-cancer, prognosis, mutation, tumor microenvironment, chemotherapy, immunotherapy

## Abstract

**Simple Summary:**

A reliable efficacy predictor for immunotherapy is warranted due to its unsatisfying responsive rate among cancer patients. Endonuclease VIII-like protein 3 (NEIL3) is a DNA glycosylase reported to be heightened in specific cancers. However, little is known regarding the immunological features of NEIL3 among cancers. Here, a pan-cancer landscape was presented to elucidate the characteristics of NEIL3 in the tumor microenvironment (TME). In this study, we demonstrated that NEIL3 may be a promising indicator for tumor prognosis, immunotherapy, and chemotherapy.

**Abstract:**

With the aim of enhancing the understanding of NEIL3 in prognosis prediction and therapy administration, we conducted a pan-cancer landscape analysis on NEIL3. The mutation characteristics, survival patterns, and immune features of NEIL3 across cancers were analyzed. Western blotting, qPCR, and immunohistochemistry were conducted to validate the bioinformatics results. The correlation between NEIL3 and chemotherapeutic drugs, as well as immunotherapies, was estimated. NEIL3 was identified as an oncogene with prognostic value in predicting clinical outcomes in multiple cancers. Combined with the neoantigen, tumor mutational burden (TMB), and microsatellite instability (MSI) results, a strong relationship between NEIL3 and the TME was observed. NEIL3 was demonstrated to be closely associated with multiple immune parameters, including infiltrating immunocytes and pro-inflammatory chemokines, which was verified by experiments. More importantly, patients with a higher expression of NEIL3 were revealed to be more sensitive to chemotherapeutic regimens and immune checkpoint inhibitors in selected cancers, implying that NEIL3 may be an indicator for therapeutic administration. Our study indicated NEIL3 has a strong association with the immune microenvironment and phenotypic changes in certain types of cancers, which facilitated the improved understanding of NEIL3 across cancers and highlighted the potential for clinical application of NEIL3 in precision medical stratification.

## 1. Introduction

Tumorigenesis is a complex process that may be described as an imbalance of cell growth and cell death, and tumor microenvironment (TME) is an indispensable key factor in its initiation [1]. The mutual effect of tumor cells and the elements of TME guarantee that undesirable cancer cells succeed in immunoediting [2,3]. Thus, it is of importance to explore the risk factors playing a part in tumor-induced immune silencing and evasion, which may provide some insights into treatment options. The association between DNA repair and cancer development has been widely investigated [4]. Genes related to the DNA repair process have demonstrated a dominant position in inducing drug resistance [5]. Accordingly, evaluation of DNA repair-related genes may better the understanding of their roles in tumorigenesis.

Endonuclease VIII-like protein 3 (NEIL3) is a member of the mammalian oxidized base-specific DNA glycosylase family. It contains a DNA binding pocket in its catalytic domain, enabling it to endure a wide spectrum of harmful effects. Previous studies have demonstrated that the tumor glyco-code process may be an essential component in tumor evasion and can serve as a novel immune checkpoint for immunotherapy [6]. Aberrant expression of glycosylase participates in immune evasion and induces immunosuppression [7,8]. In addition, glycosylase may play a role in generating neoantigens by glyco-code tumor-related proteins, indicating its possible utility in generating targets for tumor-specific T-cell responses [9]. Existing evidence suggests that NEIL3 plays a crucial role in preventing the accumulation of cytotoxic and mutagenic DNA lesions by initiating the recognition and removal of damaged bases [10]. As a glycosylase closely related to DNA repair, the role of NEIL3 among tumors may be of great importance. Previously, Zhao et al. proved that NEIL3 repairs oxidative lesions at telomeres during mitosis and prevents senescence in hepatocellular carcinoma (HCC) by using the NEIL3-depleted system [11]. Wang et al. reported that the loss of NEIL3 in cancerous cells promotes chemotherapy resistance in prostate cancer [12]. However, the function of NEIL3 as a glycosylase, as well as its immunological role in TME, is still unclear, based on recent studies. 

In the past few decades, immunotherapy has become one of the most effective and popular cancer treatment options. The application of anti-programmed cell death protein 1 (PD-1) and anti-cytotoxic T-lymphocyte-associated protein 4 (CTLA-4) agents has greatly benefited cancer patients and achieved satisfactory outcomes in clinical practice. Unfortunately, this type of therapy has been revealed to be only effective for some tumor indications [13]. The dilemma of chemotherapy resistance and immunotherapy non-response in tumors has become an increasingly severe problem facing clinicians. The reasons for drug resistance are multifactorial and include dysfunction of the immune system and cancer-promoting factors in TME [14]. Given the heterogeneity in patients’ responses to different therapies, the development of precise and personalized treatment is urgently needed. With the development of next-generation sequencing (NGS), where various bioinformatics approaches are applied to preferably match patients to drugs, emerging immune-related targets have been presented to the public, and targeted drugs have been developed [15,16]. Among these targets discovered by data mining, NEIL3 may be noteworthy. NEIL3 is increased in several tumors, such as pancreatic adenocarcinoma (PAAD), lung adenocarcinoma (LUAD), and lower-grade glioma (LGG), and it seems to be highly expressed in cells with proliferative potential [17]. The existing studies on NEIL3 mostly focused on its association with DNA repair. Nevertheless, there is no integrative study reporting the immunological features of NEIL3 among cancers. Herein, by conducting a pan-cancer analysis, this study has examined the immunological and oncogenic role of NEIL3 in TME across cancers for the first time, which may provide insight into the potential of NEIL3 in clinical strategies. 

In this study, we comprehensively investigated the NEIL3 expression level and its correlation with prognosis. In addition, immunological features comprising immunocyte infiltration, somatic mutation, DNA repair-related genes, DNA methyltransferase genes, and inflammatory expression were analyzed. Notably, we characterized the relationship between NEIL3 expression and chemotherapy drug resistance, as well as immunotherapy sensitivity, across cancers. Together, our findings have highlighted that NEIL3 may be a promising indicator for tumor immunotherapy and chemotherapy.

## 2. Materials and Methods

### 2.1. Patient Samples

Twelve pairs of fresh specimens were collected from the Third Affiliated Hospital of Sun Yat-sen University to compare NEIL3 mRNA expression between HCC and adjacent non-tumor tissues [18]. Another 24 HCC samples were collected to explore the mRNA level of NEIL3 and immune biomarkers. Tissues were stored in RNAlater solution (AM7020, Invitrogen) after surgical resection for quantification of mRNA. A total of 118 paraffin-embedded HCC specimens for prognostic survival analysis were obtained from Sun Yat-sen University Cancer Center. Commercial multi-organ tissue arrays (HOrgC120PG05) were purchased from Shanghai OUTDO Biotech Cooperation to evaluate the correlation of NEIL3 and CCR4 across cancers. This study was approved by the Institute Research Ethics Committee at the Third Affiliated Hospital of Sun Yat-sen University. Written informed consent was obtained from each patient. Relevant experiments with these samples were performed following the relevant regulations.

### 2.2. NEIL3 mRNA Expression Profiles across Cancers

The expression levels of NEIL3 in 31 normal human tissues and 21 tumor cell lines were obtained from the Genotype-Tissue Expression (GTEx) portal (GSE92743; https://gtexport.org/home/, accessed on 7 November 2020) and Cancer Cell Line Encyclopedia (CCLE) (GSE36139; https://portals.broadinstitute.org/ccle/about, accessed on 7 November 2020). The differences in NEIL3 expression between cancer and normal tissues, as well as tumor and adjacent paired tissues, were analyzed with data from The Cancer Genome Atlas (TCGA) (https://portal.gdc.cancer.gov/, accessed on 7 November 2020) [19]. R software (version 3.6.4) was used to calculate the expression differences between normal and tumor samples in each cancer type. Unpaired Wilcoxon Rank-Sum and Signed-Rank tests were used to analyze the significance of the differences. The threshold was set as follows: *p* value of 0.05 and fold change of 1.5.

### 2.3. Prognostic Analysis

We downloaded the normalized batched pan-cancer dataset, TCGA Pan-Cancer, from the UCSC (https://xenabrowser.net/, accessed on 12 December 2020) database and further extracted NEIL3 (ENSG00000109674) gene expression data in each sample. We obtained a high-quality TCGA prognostic dataset from a previously published study [20]. Samples with a follow-up period of less than 30 days and cancer types with less than 10 samples were excluded. Logarithmic transformation was performed on each expression value. Patients with different cancers were divided into two groups according to the median of the NEIL3 expression level. The impact of NEIL3 expression on patient prognosis, including overall survival (OS), progression-free interval (PFI), disease-specific survival (DSS), and disease-free interval (DFI), in a variety of cancers was all analyzed by the Cox proportional hazards regression model. Forest plots were generated, and the hazard ratio with a 95% confidence interval and log-rank *p* value were calculated.

### 2.4. Cell Culture

Immortalized human hepatic cell lines (MIHA and LO2), HCC cell lines (C3A, LM3, Huh-7, PLC/PRF/5, MHCC-97H, HepG2, HepG2.215, SNU-449, SK-Hep1, QGY-7703, Hep3B, SNU-449, BEL-7405), immortalized colonic epithelial cells (HCOEPIC), colon adenocarcinoma (COAD) cell lines (HCT116, HT29, RKO, SW480), immortalized renal cortical proximal convoluted tubular epithelial cells (HK2), and kidney renal clear cell carcinoma (KIRC) cell lines (769P, 786O) were utilized in this study and cultured in Dulbecco’s modified Eagle’s medium (DMEM, Gibco) or RPMI 1640 medium (1640, Gibco) containing 10% fetal bovine serum (FBS, Gibco) at 37 °C and 5% CO2. All hepatic and HCC cell lines were harvested from the College of Life Sciences, Sun Yat-sen University. The HCOEPIC and COAD cell lines were gifts from Sun Yat-sen University Cancer Center, and HK2 and KIRC cell lines were gifts from the Department of Urology Surgery, the Third Affiliated Hospital of Sun Yat-sen University. Cells were cultured and passaged as previously described [18].

### 2.5. Western Blotting

Cell lysis, the measurement of lysate protein concentration, and Western blotting were conducted as previously described [21]. Primary Antibody Dilution Buffer (P0023A, Beyotime) was used to dilute primary antibodies, including NEIL3 (GTX55960, GeneTex, 1:1000), β-actin (60008-1-Ig, Proteintech, 1:5000), and GAPDH (D16H11, CST, 1:1000).

### 2.6. Reverse Transcription and Quantitative PCR (RT-qPCR)

Total RNA was isolated from cell lines and tissue specimens using TRIzol reagent (Invitrogen) according to the manufacturer’s protocol. Total RNA (2 µg) was reverse transcribed into cDNA, and qPCR was performed according to the previous description [21]. The information on the primers is described in Supplementary Table S1.

### 2.7. Immunohistochemistry

All paraffin-embedded HCC samples were cut into 4-μm sections on a glass slide. These slides and the commercial multi-organ tissue arrays were dried overnight at 37 °C and then sequentially placed into xylene and graded alcohol hydrogen peroxide. Next, the deparaffinized sections were subjected to heat-mediated antigen retrieval in ethylenediaminetetraacetic acid (EDTA) solution (pH 8.0). The primary antibodies anti-NEIL3 (GTX55960, GeneTex, 1:400), and anti-CCR4 (DF10206, Affinity, 1:200) were used to incubate the samples, followed by the secondary antibody (K5007, Dako). The expression score was defined as the intensity score (0–3 points) multiplied by the extent score (0–4 points).

### 2.8. NEIL3 Mutational Feature Analysis

Mutation information of NEIL3 was visualized by the cBioPortal database (https://www.cbioportal.org/, accessed on 4 January 2021) based on data extracted from the TCGA pan-cancer atlas. Data consisting of structural variants, mutations, and copy number alterations (CNAs) were analyzed, and the NEIL3 alteration frequency across cancers was assessed. A 3D structure plot and mutation diagram were used to describe the basic construction of the NEIL3 molecule. The association between NEIL3 mutation and OS across cancers was also explored by cBioPortal. Gene Set Variation Analysis (GSVA) was performed to compare the differences in enriched pathways between the NEIL3 mutation and non-mutation groups. Wilcoxon Rank-Sum and Signed-Rank Tests were used to analyze the expression of NEIL3 mutations and non-mutation across cancers. The altered expression of tumor-related genes in samples harboring NEIL3 mutation was evaluated on the MuTARGET website (https://www.mutarget.com/, accessed on 4 January 2021). Functional changes in NEIL3 with certain site mutations were predicted by the PolyPhen-2 tool (http://genetics.bwh.harvard.edu/pph2/, accessed on 21 May 2021). The association between NEIL3 mutation and immune checkpoint genes was evaluated using the Tumor Immune Estimation Resource database (TIMER) (http://timer.cistrome.org/, accessed on 4 January 2021).

### 2.9. Pan-Cancer Grade and Stage Analysis

Raw clinical information of patients with different cancers was acquired from the TCGA database and entered into the Tumor-immune System Interactions Database (TISIDB) (http://cis.hku.hk/TISIDB/, accessed on 18 January 2021), an integrated repository portal for tumor-immune system interactions to facilitate classification of patients into different subgroups according to pathological grades and stages, which is determined by AJCC principles.

### 2.10. Tumor Immune Infiltration Analysis

We utilized the TIMER database to investigate the relationship between 6 types of immunocyte infiltration (B cells, CD4 T cells, CD8 T cells, neutrophils, macrophages, and dendritic cells (DCs)) and NEIL3 expression, as well as mutation. In addition, ESTIMATE algorithms (version 1.0.13) were used to assess the proportion of tumor-related immunocyte infiltration and the amount of immune and stromal components from the TCGA database. To identify significantly correlated immune infiltration scores, we calculated the Pearson’s correlation coefficient between NEIL3 expression and immune infiltration scores in individual cancer types using the psych R package. All the gene expression levels were expressed as log2 RSEM.

### 2.11. Immune-Related Biomarker and Immune Subtype Analysis

Immune checkpoint genes, as well as immune-related biomarkers, including immuno-inhibitors, immuno-stimulators, MHC molecules, chemokines, chemokine receptors, and lymphocytes, were obtained from The Cancer Immunome Atlas database (https://tcia.at/home, accessed on 5 February 2021). The correlation between NEIL3 expression and immune-related biomarkers was assessed through TISIDB. A heatmap and scatter plot were drawn to show their relationship. The distribution of NEIL3 expression with different immune subtypes across various cancers was obtained from TCGA datasets. 

### 2.12. Tumor Neoantigen, Mutational Burden (TMB), and Microsatellite Instability (MSI) Analysis

NEIL3 expression data were extracted from the TCGA Pan-Cancer dataset. MSI score and neoantigen data of each cancer type were obtained from the previous study [22,23]. R package maftools was used to evaluate TMB of each cancer type. Pearson correlation analysis was conducted to study the relationship between NEIL3 expression and neoantigen, TMB, and MSI.

### 2.13. Mismatch Repair (MMR) Gene Mutation, DNA Methyltransferase, EMT Pathway Activity, and Gene Set Enrichment Analysis (GSEA)

The mutation levels of DNA MMR genes (MLH1, MSH2, MSH6, PMS2, and EPCAM) and the expression levels of 4 DNA methyltransferase genes in tumors were acquired from the TCGA database. The association between NEIL3 expression and EMTpathway activity was analysed by the GSCA website tool (http://bioinfo.life.hust.edu.cn/GSCA/, accessed on 17 February 2021). Analysis of the NEIL3 related pathways was performed via the GSEA website (https://www.gsea-msigdb.org/gsea/, accessed on 17 February 2021).

### 2.14. Chemotherapy and Immunotherapy Response Analysis

Data on NEIL3 expression and chemotherapy drugs were derived from the Cancer Genome Project (CGP), Cancer Therapeutics Response Portal (CTRP), and Cancer Cell Line Encyclopedia (CCLE) databases. The correlation between NEIL3 expression level and chemotherapeutic drugs was estimated by Computational Analysis of Resistance (CARE) algorithms. The top 10 chemotherapeutic drugs associated with NEIL3 expression are presented as a scatter plot. CARE scores were utilized to indicate the association between NEIL3 molecular alteration and drug efficacy. A positive score indicates a higher expression value (or presence of mutation) associated with drug response. TISIDB was used to analyze NEIL3 expression and mutation profiles among responders and non-responders who received immunotherapy.

### 2.15. Statistical Analysis

The Kruskal–Wallis test was utilized to investigate NEIL3 expression levels in various tumor tissues and cancer cell lines. The difference in NEIL3 expression levels in tumor and normal tissues was assessed by the t-test. In the survival analysis, the hazard ratios and *p* values were computed by the univariate Cox regression method. The relationship between NEIL3 expression and immunocyte infiltration levels was analyzed via the Spearman correlation method. Pearson correlation analysis was utilized to evaluate the relationship between the expression levels of NEIL3 and immune infiltration scores, tumor neoantigen, TMB, MSI genes, MMR genes, and DNA methyltransferase genes. The significance threshold of all statistical analyses was *p* < 0.05. 

## 3. Results

### 3.1. NEIL3 Expression Is Comparably Increased in Tumor Cells or Tissues across Cancers

**Figure 1:**
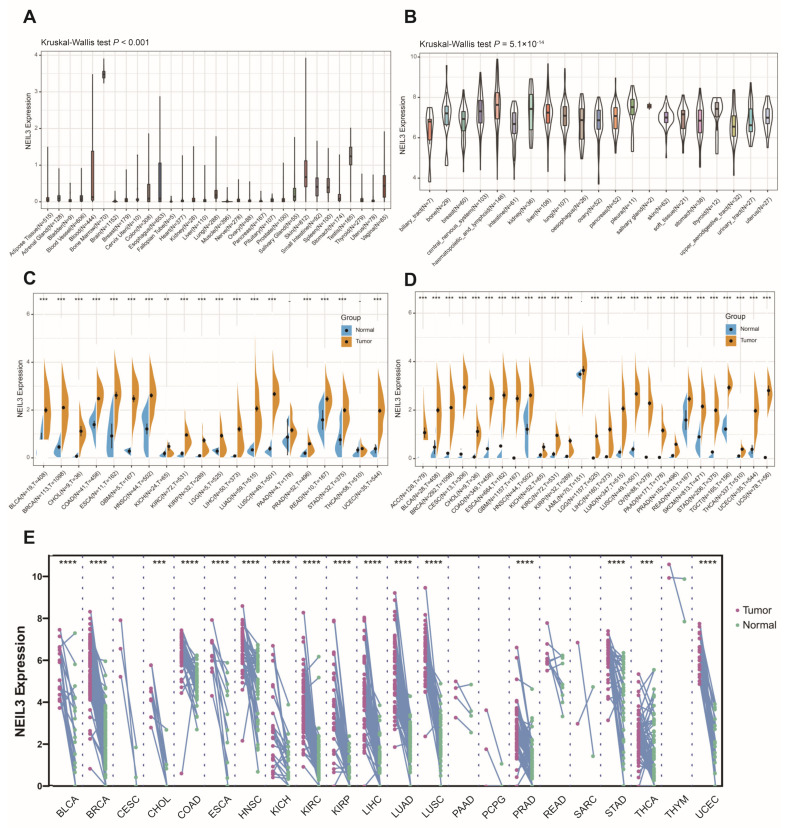
The mRNA expression level of NEIL3 across human cancers. The mRNA expression of NEIL3 in (**A**) 31 types of normal tissues (N, number of samples); (**B**) 21 types of cancer cell lines in CCLE database (N, number of samples); (**C**) 20 types of cancers and normal tissues in TCGA database (N, normal; T, tumor); (**D**) tumor and normal tissues in TCGA combined with GTEx database (N, normal; T, tumor); and (**E**) tumor and paired adjacent normal tissue in TCGA database. ** *p* < 0.01, *** *p* < 0.001, **** *p* < 0.0001.

The analysis revealed that NEIL3 expression was generally low in normal tissues, except bone marrow (Figure 1A), and upregulated in all 21 types of tumor cell lines (Figure 1B). In addition, it was significantly higher in tumors than in normal tissues (Figure 1C), which was confirmed in TCGA combined with the GTEx database (Figure 1D). The results of the paired tumor and adjacent cancerous samples showed that NEIL3 was heightened in 16 types of cancers, with sufficient paired samples at mRNA level (Figure 1E).

**Figure 2:**
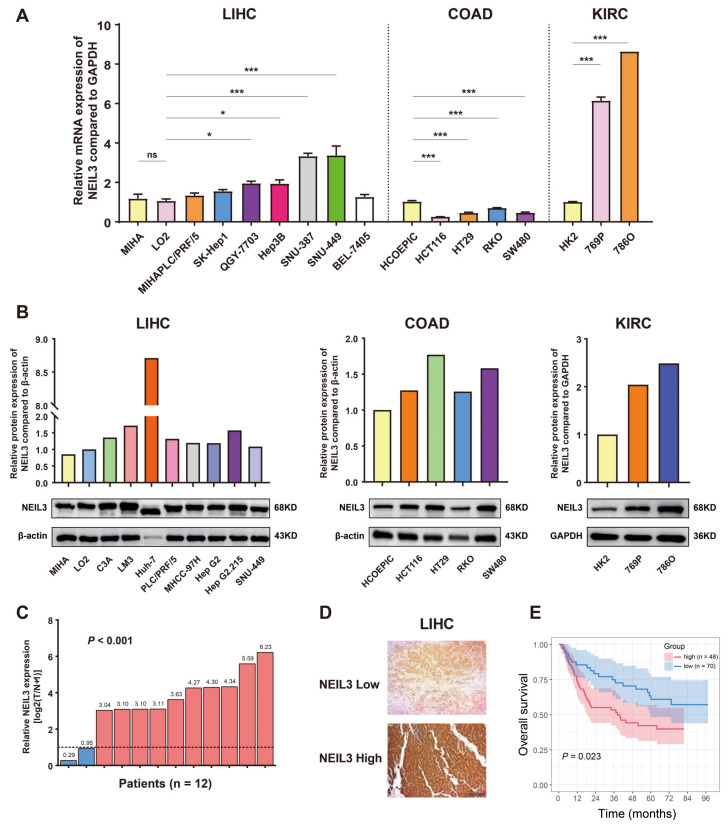
Experimental validation of the expression and prognosis of NEIL3 in cancers. (A) mRNA expression of NEIL3 in LIHC, COAD, and KIRC cell lines. (B) Protein expression of NEIL3 in LIHC, COAD, and KIRC cell lines, as well as their quantitative results. (**C**) mRNA expression of NEIL3 in 12 patients’ LIHC and paired adjacent liver tissues. (**D**) Representative images of IHC staining of HCC patient samples with low and high NEIL3 expression. (**E**) Difference in OS between high and low NEIL3 expression groups. ns indicates no significance, * *p* < 0.05, *** *p* < 0.001.

Next, we confirmed the above findings using in vitro cancer cell lines of different origins. Compared with normal hepatic LO2 and MIHA cells, the mRNA expression of NEIL3 was remarkably upregulated in HCC cell lines (Figure 2A). We also validated these findings at the protein expression level and noticed that NEIL3 was also increased in C3A, LM3, PLC/PRF/5, HepG2.215, MHCC-97H, and especially Huh-7 cells (Figure 2B). Similarly, the mRNA and protein expressions of NEIL3 were both increased in KIRC cell lines (769P, 786O) compared with the normal control HK2 cells. Notably, we discovered that NEIL3 expression was upregulated in COAD cancer cells at the protein level, but reduced at the mRNA level (Figure 2A,B). Consistent with the above results, our experiment conducted in HCC validated that NEIL3 mRNA expression was significantly upregulated in HCC tumor compared with adjacent normal tissue (10 in 12 pairs) (Figure 2C). It was also increased in HCC tissues compared with normal tissues at protein level (Figure S1A). Taken together, the results indicated that NEIL3 is abnormally heightened in a majority of cancers, indicating its possibility as an oncogene.

### 3.2. NEIL3 Expression Possesses Prognostic Value in Cancers

We detected an upregulation of NEIL3 levels in higher grades in cancers such as KIRC, LGG, LIHC (liver hepatocellular carcinoma), PAAD, uterine corpus endometrial carcinoma (UCEC), and head and neck squamous cell carcinoma (HNSC) (Figure S2). NEIL3 expression was also positively related to cancer stage, such as adrenocortical carcinoma (ACC), kidney chromophobe (KICH), KIRC, kidney renal papillary cell carcinoma (KIRP), LIHC, LUAD, and lung squamous cell carcinoma (LUSC) (Figure S3).

Notably, the high expression level of NEIL3 was significantly related to shorter DFI in most cancers (Figure S4A–G,I), while it was the opposite in stomach adenocarcinoma (STAD) (Figure S4H). Since non-tumour-related factors can lead to death during the follow-up period, the correlation between NEIL3 expression and DSS was analyzed in 33 cancers and is shown in Figure S5A. Elevated NEIL3 expression was significantly correlated with lower DSS in 13 cancers (Figure S5B–N). In addition, the correlation between NEIL3 expression and patient OS, as well as PFI, was also analyzed in 33 cancers and is shown in Supplementary Figure S6 and Figure S7, with the results nearly the same as those obtained from DSS analysis.

Following the bioinformatics analysis, we collected 118 HCC patient samples to validate the prognostic role of NEIL3 (Supplementary Table S2) and categorized them into high or low NEIL3 expression group (Figure 2D). The survival curves generated by Kaplan–Meier survival analysis showed that HCC patients with high NEIL3 expression (n = 48) had significantly shorter OS than those with a low NEIL3 level (n = 70) (*p* = 0.023) (Figure 2E). Furthermore, univariate and multivariate Cox regression analyses were performed according to sex, age, AFP, grade, stage, and NEIL3 expression. The result revealed that NEIL3 may be an independent OS indicator in HCC (Figure S1B).

Generally, higher NEIL3 expression predicts poorer prognosis in cancer patients, suggesting its possibility as a prognostic indicator.

### 3.3. NEIL3 Presented a High Mutation Frequency among Cancers

**Figure 3:**
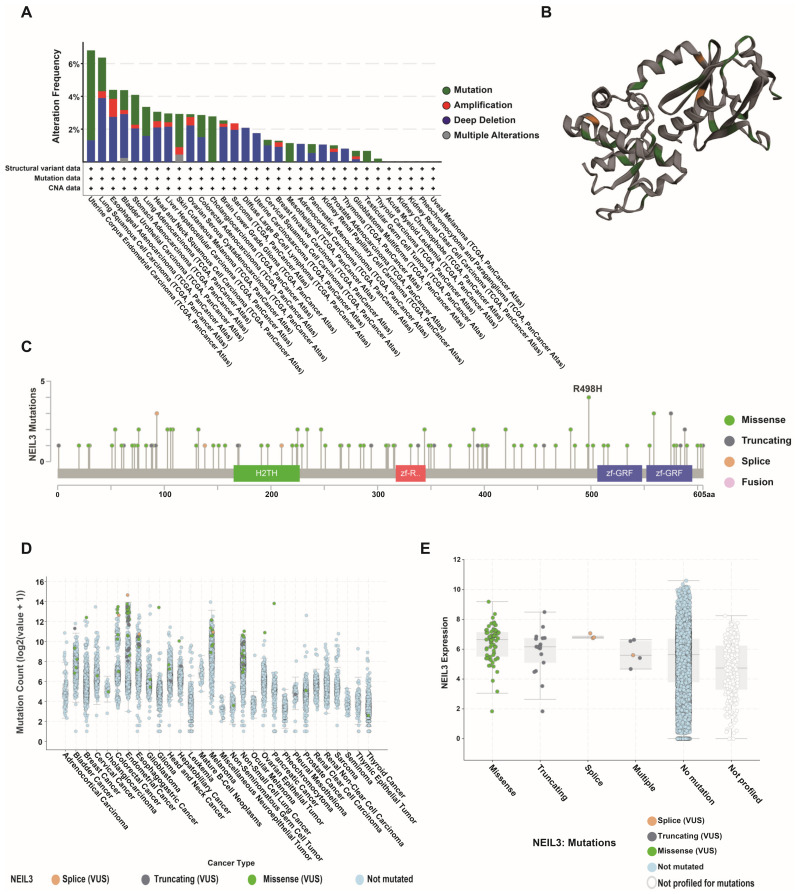
Mutation features of NEIL3 among cancers. (**A**) Alteration frequency of NEIL3 among 33 types of cancers. (**B**) Three-dimensional structure diagram of the NEIL3 protein. The green part represents the missense mutation area, black represents the truncating mutation area, and orange represents the inframe mutation area. (**C**) Mutation diagram of NEIL3. Light green circles represent missense mutations, light orange circles represent splice mutations, and gray circles represent truncating mutations. In the case of different mutation types at a single position, the color of the circle represents the most frequent mutation type. (**D**) Fractional genomic alteration of NEIL3 across 32 cancers. Each dot represents a sample. (**E**) Mutation types of NEIL3 at mRNA expression level.

NEIL3 exhibited a wide range of genomic alterations among various types of cancers. In total, 27 cancers presented NEIL3 alterations, whereas only 5 cancers showed no alterations. The alterations of NEIL3 are of high frequency and exhibit different patterns. Gene mutation and deep deletion were the main alterations. In particular, these two types of alterations have a prominently high prevalence in UCEC and LUSC (Figure 3A). As a glycosylase, NEIL3 is composed of four domains, namely, a formamidopyrimidine-DNA glycosylase H2TH domain, a Zn-finger in Ran binding protein (Zf-R) domain, and two GRF zinc finger (zf-GRF) domains. Mutations were dispersed throughout the protein-coding chain (Figure 3B,C). Concerning the fractional genome alteration of NEIL3 across cancers, the main mutation type was mostly missense mutations (Figure 3D). Collectively, NEIL3 presented a high mutation frequency among cancers.

### 3.4. Altered NEIL3 May Have an Impact on Tumors

**Figure 4:**
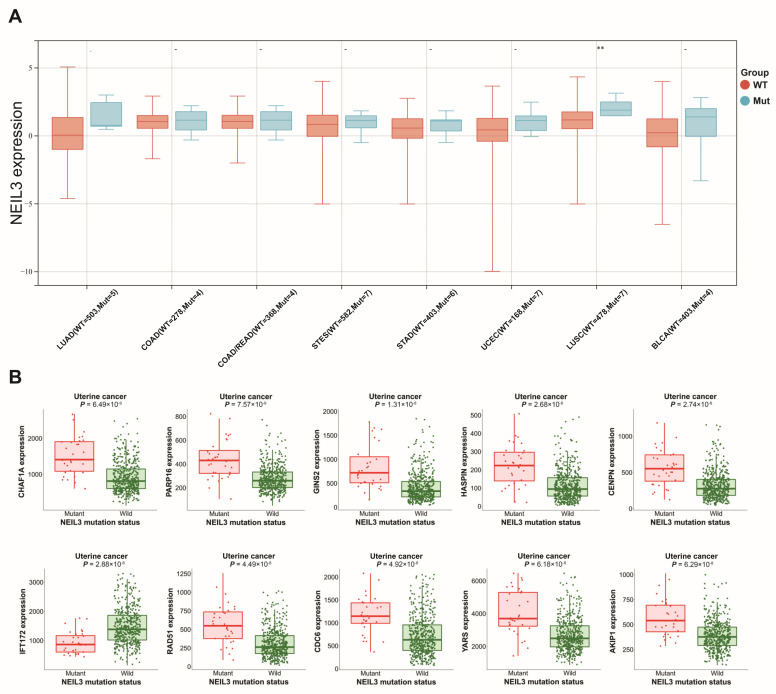
Prediction of NEIL3 expression and affected genes related to NEIL3 mutation. (**A**) The expression of NEIL3 between NEIL3 mutation and non-mutation groups. (**B**) Expression of the genes affected by NEIL3 mutation. ** *p* < 0.01.

Patient samples from TCGA with NEIL3 mutation information were included to evaluate the expression difference in NEIL3 between the mutation and non-mutation groups. The results showed that NEIL3 was only increased in the mutation group in LUSC compared with the non-mutation group, while no significant difference in expression was found in LUAD, COAD, colon adenocarcinoma/rectum adenocarcinoma esophageal carcinoma (COADREAD), stomach and esophageal carcinoma (STES), STAD, UCEC, and bladder urothelial carcinoma (BLCA) (Figure 4A). 

**Figure 5:**
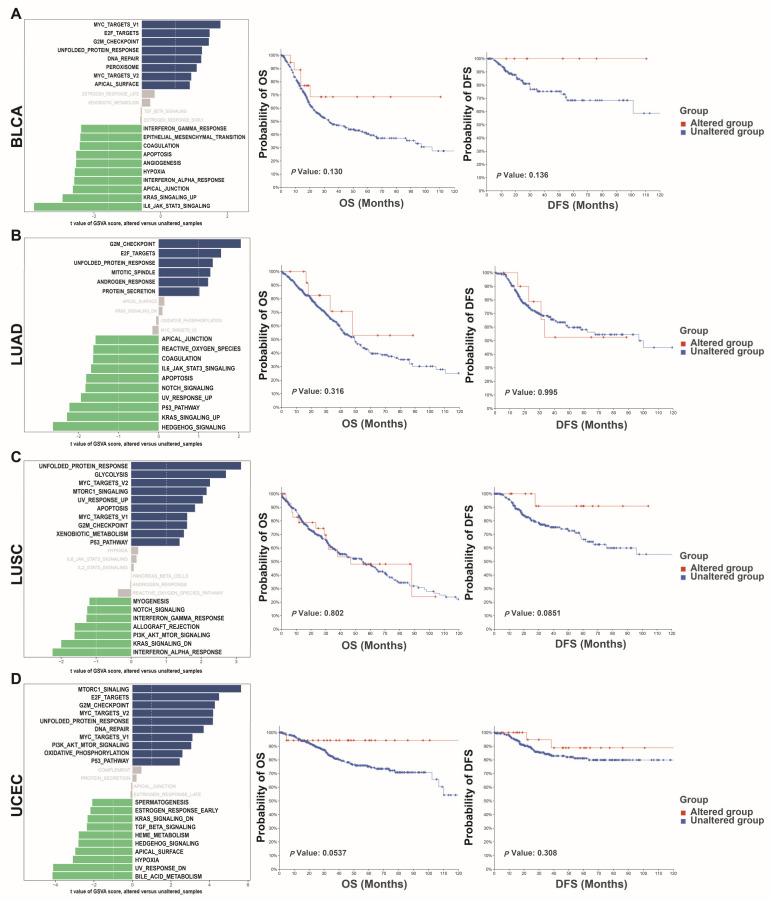
Potential role of altered NEIL3 in cancers. (**A**–**D**) GSVA of enriched pathways and OS analysis of the NEIL3-altered and -unaltered groups in BLCA (**A**), LUAD (**B**), LUSC (**C**), and UCEC (**D**).

Survival analysis revealed that no significant difference existed between the NEIL3-altered and -unaltered groups. To explore the potential effect of NEIL3 mutation in cancers, GSVA was conducted. Altered NEIL3 may affect the G2/M checkpoint pathway in BLCA, LUAD, LUSC, and UCEC (Figure 5A–D). In addition, the results from MuTARGET showed that the expression of CHAF1A, PARP16, GINS2, HASPIN, CENPN, IFT172, RAD51, CDC6, YARS, and AKIP1 was upregulated in the NEIL3-mutated groups (Figure 4B). Meanwhile, the results generated by the PolyPhen-2 tool indicated that NEIL3 mutation at amino acid positions 103, 106, 344, 481, and 498 was benign, but probably damaging at amino acid positions 76, 96, 132, 225, 234, 247, 390, and 559 (Table 1). 

Next, we studied the relationship between NEIL3 mutation and immune checkpoint genes. The results showed that NEIL3 mutation is positively associated with the expression of CD274, CD80, CTLA-4, HAVCR2, ICOS, LAG3, PDCD1, PDCD1LG2, TIGIT, TNFRSR9, and TNFSF9 in UCEC. A positive relationship can also be detected between NEIL3, CTLA-4, ICOS, LAG3, and TIGIT in BRCA-LumA (Figure S8).

### 3.5. NEIL3 Contributes to Immune Infiltration among Cancers

**Figure 6:**
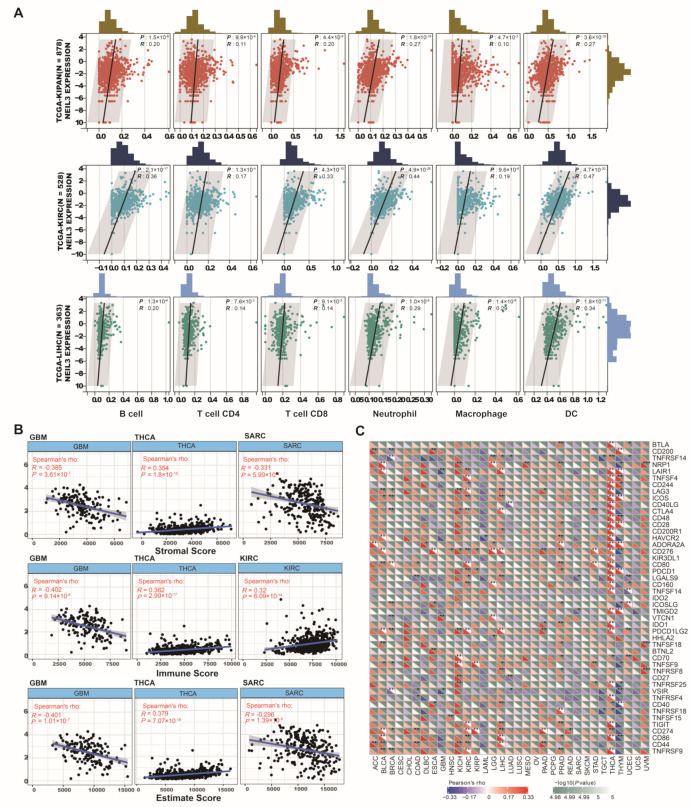
NEIL3 expression level is correlated with immune characteristics in cancers. Correlation between the expression level of NEIL3 and (**A**) lymphocyte infiltration in KIPAN, KIRC, and LIHC; (**B**) stromal score, immune score, and ESTIMATE score of the top 3 cancers; and (**C**) 40 common immune checkpoint genes across cancers. * *p* < 0.05, ** *p* < 0.01, *** *p* < 0.001.

As the grade of tumor-infiltrating lymphocytes is valued as an independent predictor of patient prognosis [24], immunocyte infiltration, along with diverse NEIL3 expression, was investigated in 33 cancer types. NEIL3 showed a positive correlation with six types of immune cells in three cancer subgroups (pan-kidney cancer cohort (KIPAN, including KICH, KIRC, and KIRP), KIRC, and LIHC), involving B cells, CD8 T cells, CD4 T cells, DCs, macrophages, and neutrophils (Figure 6A). Notably, a positive correlation existed in 5 out of 6 types of immune cell infiltration in thyroid carcinoma (THCA), prostate adenocarcinoma (PRAD), LGG, and BLCA (Figure S9).

The relative fraction of cell components is helpful to describe the immune characteristics of TME. Hence, we utilized the immune score (Figure S10), ESTIMATE score (Figure S11), and the stromal score (Figure S12) to further quantify the level of immune infiltration across cancers. Upregulated NEIL3 expression was significantly positively correlated with the stromal score of THCA and negatively correlated with that of GBM (glioblastoma multiforme) and SARC (sarcoma) (Figure 6B). Similarly, a positive correlation exists between NEIL3 expression and the immune score of THCA and KIRC and the ESTIMATE score of THCA, despite a negative correlation regarding the immune score of GBM or the ESTIMATE score of GBM and SARC. 

**Figure 7:**
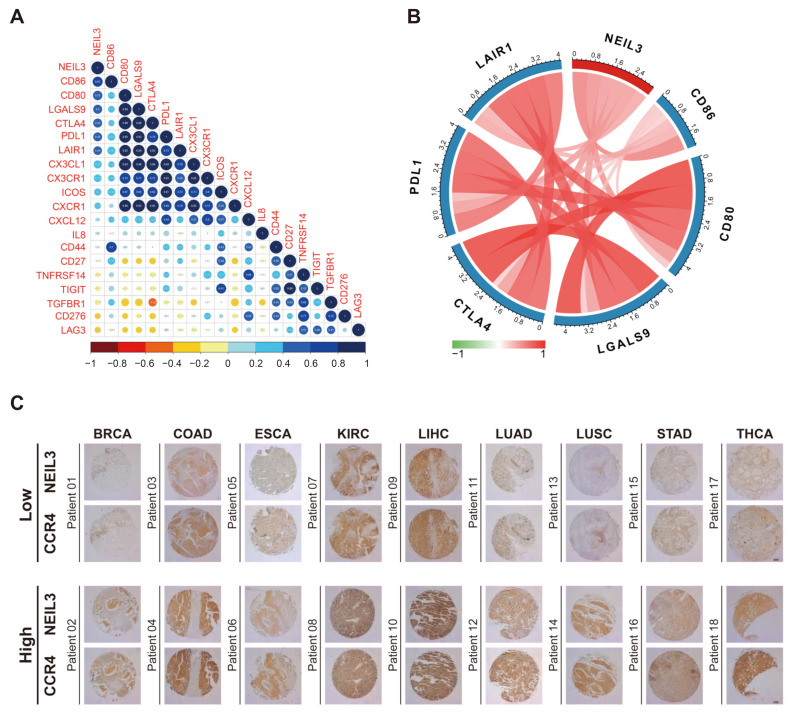
Experimental validation of the association of NEIL3 and immunity in cancers. (**A**) mRNA expression of NEIL3 and immune biomarkers in 24 LIHC samples. (**B**) Immune biomarkers positively correlated with NEIL3 expression with statistical significance. (**C**) Representative images of IHC staining for NEIL3 and CCR4 in 9 types of cancers.

Immune checkpoint inhibitors (ICIs), as ever-expending agents, occupy an essential position in cancer immunotherapy [25]. Therefore, the correlation between NEIL3 expression and over 40 common immune checkpoint genes was analyzed (Figure 6C). Strikingly, in THCA and THYM (thymoma), NEIL3 was significantly associated with more than 20 immune checkpoint genes, such as CD86, ICOS, TNFSF4, and CD48, implying its role in regulating the tumor immune microenvironment. Our experimental results validated that NEIL3 is positively associated with the expression of CD86, CD80, LGALS9, CTLA-4, PDL1, and LAIR1 in HCC samples (Figure 7A,B). Overall, the results mentioned above indicated that NEIL3 has a strong correlation with immune checkpoint genes. 

### 3.6. NEIL3 Has a Close Relationship with Multiple Immune Biomarkers of Cancers

Regarding the immunosuppressive genes, NEIL3 is largely correlated with their upregulated expression in KIRC, KIRP, and LGG (Figure S13A), while presenting a negative correlation regarding immunostimulatory genes in testicular germ cell tumors (TGCT), UCEC, and GBM (Figure S13B). Specifically, NEIL3 expression was significantly associated with LAG3 in KIRC and TMEM173 in LUAD. Genes coding MHC molecules are also essential in the immune response against intracellular pathogens [26]. MHC class II (MHCII) can be conditionally expressed in tumor cells, which is of great significance for antitumour immunity [27]. Along with increased NEIL3 expression, the expression of most MHCII-encoding genes was decreased in most cancers (Figure S13C). Notably, NEIL3 had an opposite relationship with HLA-DQA2 in KICH.

Chemokines serve as communication for immune cells and non-immune cells [28]. Various chemokines have different effects on tumor immunity [29]. Pro-tumor chemokines, such as CXCL8, CXCL9, CXCL10, and CXCL11, can promote cancer cell proliferation and metastasis [30], and our results revealed that NEIL3 expression was positively correlated with them. Metastasis is the prominent feature of advanced cancers [31], and NEIL3 was correlated with many metastasis-inducing chemokines, such as CXCL10, which confirms our previous result that NEIL3 is related to an advanced stage and poorer prognosis in cancers. On the contrary, anti-tumour chemokines, such as CCL14 and CX3CL1 [32], were related to low NEIL3 expression in most cancers (Figure S13D). Chemokine receptors, as signal transducers for chemokines, also function diversely in cancers. In our analysis, NEIL3 expression was positively correlated with pro-tumour chemokine receptors, such as CCR1, to some extent, but negatively correlated with anti-tumour receptors, such as CX3CR1, across cancers [33] (Figure S13E).

Our analysis showed that NEIL3 expression was negatively correlated with Th1 and Th17 cells and plasmacytoid DCs (pDCs), which are immunocytes that indicate a good prognosis in cancers [34,35,36]. In contrast, NEIL3 expression was significantly associated with active CD4 T cells and Th2 cells, which implies cancer recurrence in most tumors [37] (Figure S13F). CCR4 is a biomarker expressed at the surface of Th2 cells [38]. Our IHC staining of CCR4 and NEIL3 in multi-organ tumor arrays suggested that NEIL3 is positively associated with the expression of CCR4 in BRCA, COAD, ESCA, KIRC, LIHC, LUAD, LUSC, STAD, and THCA samples (Figure 7C).

### 3.7. NEIL3 Exhibited Diverse Expression Patterns among Different Immune Subtypes across Cancers

Based on the multiple immune TME characterizations, six immune subtypes were identified: wound healing (C1), IFN-gamma dominant (C2), inflammatory (C3), lymphocyte depleted (C4), immunologically quiet (C5), and TGF-b dominant (C6) [23]. Among these immune subtypes, C1, C2, C4, and C5 were associated with high tumor proliferation or high M2 macrophage infiltration, yet C3 was associated with the best prognosis. NEIL3 expression appears to be increased in the C1, C2, and C4 subtypes of most cancers. In detail, the only immune subtype of GBM with NEIL3 expression is C4. Regarding TGCT and UCS, the major subtypes are C1 and C2. Notably, there were negative correlations between NEIL3 expression and the C3 immune subtype in most types of cancers (Figure S14). 

### 3.8. NEIL3 Is Involved with Neoantigen, TMB, and MSI in Various Cancers

**Figure 8:**
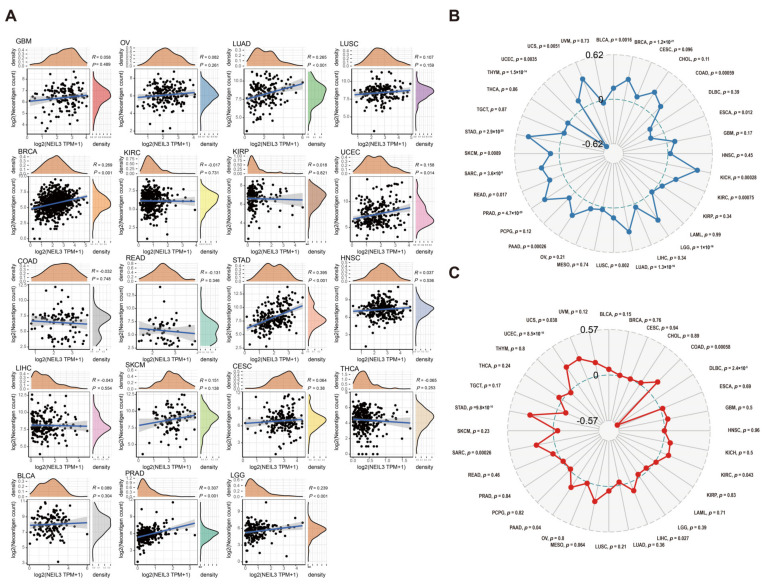
NEIL3 expression is correlated with neoantigen number, TMB, and MSI in various cancers. Correlation between NEIL3 expression level and (**A**) neoantigen number; (**B**) TMB; and (**C**) MSI across cancers.

Tumor neoantigens are mutated peptides capable of inducing T-cell antitumour responses [39]. Previous studies have shown that neoantigens are a promising target for immunotherapy [40,41,42]. Thus, we investigated the correlation of NEIL3 expression and neoantigen levels across cancers (Figure 8A). Higher NEIL3 expression was significantly correlated with richer neoantigen levels in LUAD, BRCA, STAD, PRAD, and LGG. Moreover, NEIL3 expression was positively correlated with TMB, especially in UCS, STAD, PRAD, and KICH. Interestingly, NEIL3 expression was significantly negatively correlated with TMB in THYM (Figure 8B). 

MSI signifies the hypermutator phenotype in short single nucleotide substitution and repetitive DNA sequences [43]. Cancers with a defective DNA MMR may be associated with a poorer cancer prognosis [44]. The relationship between the level of NEIL3 expression and pan-cancer MSI is presented in Figure 8C. NEIL3 expression was positively correlated with MSI. Together, the results demonstrate that the association between NEIL3 expression and neoantigen burden, TMB, and MSI was cancer dependent, implicating that NEIL3 may correlate with some factors that tend to increase responsiveness to checkpoint blockade therapy in a small subset of tumors, which provides a clue for clinical practice.

### 3.9. NEIL3 Expression Is Correlated with MMR, DNA Methyltransferase Genes, and EMT Pathway Activity across Cancers

**Figure 9:**
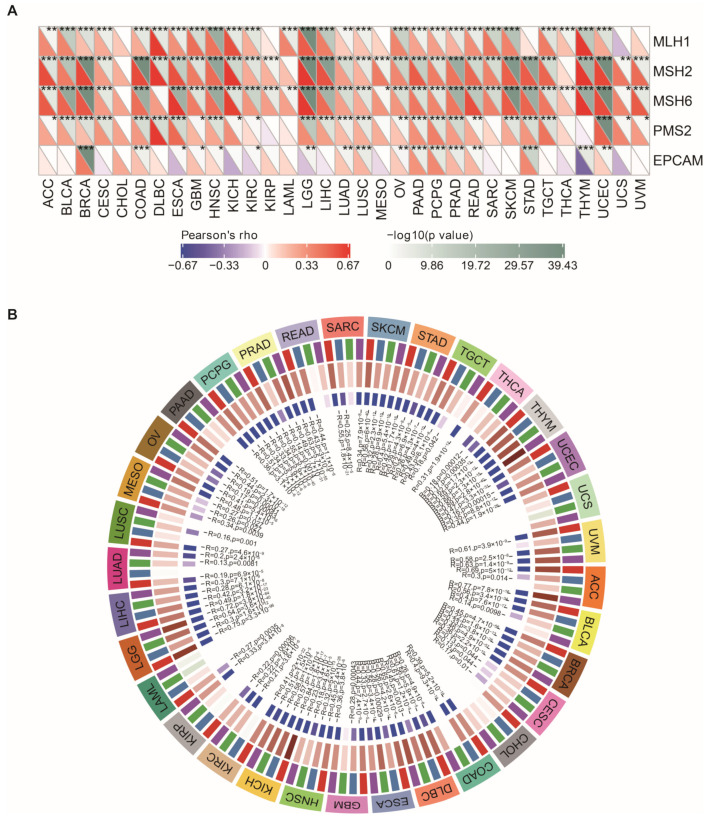
NEIL3 expression is correlated with MMR and DNA methyltransferase genes in multiple cancers. Correlation between NEIL3 expression level and (**A**) mutation of five MMR genes, namely, MLH1, MSH2, MSH6, PMS2, and EPCAM; (**B**) expression of four DNA methyltransferase genes, namely, DNMT1 (red), DNMT2 (blue), DNMT3A (green), and DNMT3B (purple), in various cancers. The innermost brown and blue circles represent the R and *p* values of the correlation analysis, respectively. * *p* < 0.05, ** *p* < 0.01, *** *p* < 0.001.

The MMR system is the mechanism underlying DNA damage repair [43]. MSI is one of the consequences of MMR deficiency. For this reason, we evaluated the relationship between NEIL3 expression and the mutation levels of five critical MMR genes across cancers. NEIL3 expression was significantly related to all or part of the five MMR genes in pan-cancer, except for CHOL (Figure 9A).

Changes in DNA methylation are considered promising targets for cancer diagnosis and prognosis [45]. The correlation between the expression level of NEIL3 and four DNA methyltransferase genes was explored. Remarkably, NEIL3 expression was positively correlated with all or part of the expression levels of these four DNA methyltransferase genes in pan-cancer, except for CHOL (Figure 9B). 

Endothelial mesenchymal transition (EMT) is a crucial step in tumor progression and metastasis. The correlation between the expression level of NEIL3 and EMT pathway activity was explored. Our results showed that higher expression of NEIL3 is positively correlated with higher EMT pathway activity in BLCA, LIHC, and THCA, but negatively in BRCA and STAD (Figure S15). Generally, NEIL3 is positively correlated with DNA MMR genes and methyltransferase expression in a majority of tumors, and the association between NEIL3 expression and EMT pathway activity is tumor-specific.

### 3.10. GSEA of Different NEIL3 Expression Levels

Upregulated NEIL3 expression was related to the downregulation of the cell cycle, oocyte meiosis, and pyrimidine metabolism pathways in the Kyoto Encyclopedia of Genes and Genomes (KEGG) gene set collection (Figure S16A), while in the HALLMARK gene set, it was related to the MTORC1 signaling, MYC targets, and E2F targets pathways (Figure S16C). Additionally, downregulated NEIL3 expression was correlated with the upregulation of aldosterone-regulated sodium reabsorption, hematopoietic cell lineage, and the intestinal immune network for IgA production in KEGG (Figure S16B), while in the HALLMARK gene set, it was correlated with bile acid metabolism, KRAS signaling, myogenesis, and coagulation (Figure S16D). Taken together, GSEA revealed that NEIL3 is related to the cell cycle, oocyte meiosis, pyrimidine metabolism, and intestinal immune network for IgA production.

### 3.11. NEIL3 May Be a Novel Indicator for Chemotherapy and Immunotherapy

**Figure 10:**
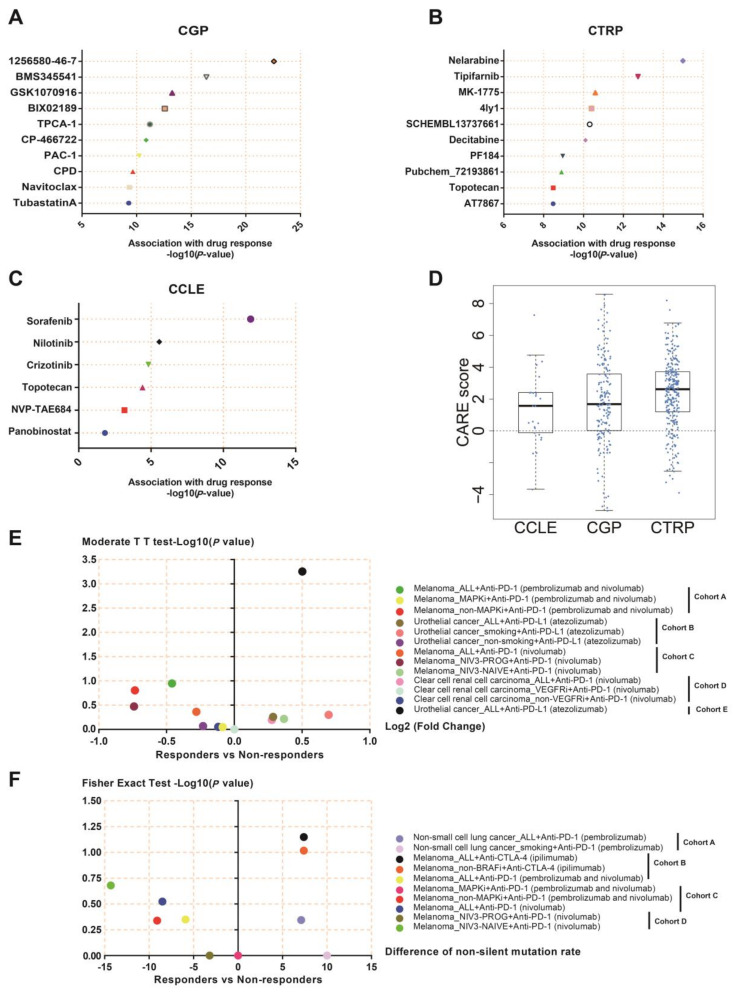
NEIL3 was correlated with chemotherapy sensitivity and ICB response. The association of NEIL3 expression level with chemotherapy sensitivity in (**A**) CGP; (**B**) CTRP; and (**C**) CCLE databases. (**D**) The CARE score of the relationship of NEIL3 and chemotherapy resistance in three databases. (**E**) The NEIL3 expression ratio between ICI responders and non-responders in the listed cohorts. Cohort A: Willy Hugo (PMID:26997480); Cohort B: Alexandra Snyder (PMID:28552987); Cohort C: Nadeem Riaz (PMID:29033130); Cohort D: Diana Miao (PMID:29301960); Cohort E: Sanjeev Mariathasan (PMID:29443960). (**F**) The difference in NEIL3 non-silent mutation rate between ICI responders and non-responders in the following cohorts. Cohort A: Naiyer A Rizvi (PMID:25765070); Cohort B: Eliezer M Van Allen (PMID: 26359337); Cohort C: Willy Hugo (PMID:26997480); Cohort D: Nadeem Riaz (PMID:29033130). 1256580-46-7, also known as Alectinib.

Our results showed that the targeted chemotherapy was effective in cancer patients with higher NEIL3 expression, especially 1256580-46-7 in the CGP database (Figure 10A), nelarabine in the CTRP database (Figure 10B), and sorafenib in the CCLE database (Figure 10C). The association of NEIL3 expression and these three compounds indicated that NEIL3 may be a reliable druggable gene that synergizes with other therapeutic biomarkers. Additionally, panobinostat, tubastatin A, and 4ly1, which are targeted regimens for histone deacetylase, may behave well in populations with NEIL3 upregulation. Moreover, the output CARE score reflected that a higher expression value of NEIL3 was associated with overall drug response (Figure 10D), which may provide insight into medication stratification.

Given the inseparable connection of NEIL3 with TME, NEIL3 expression differences between immunotherapy responders and non-responders were analyzed. NEIL3 expression in atezolizumab responders was far higher than that in non-responders with urothelial cancer. NEIL3 expression was elevated in smoking urothelial cancer patients with an atezolizumab response, although no statistical significance exists due to small sample sizes. Likewise, the melanoma patient’s response to the first administration of nivolumab revealed a higher expression of NEIL3 (Figure 10E). Based on the former results, NEIL3 is an oncogene with a high mutation rate. Although the difference in the NEIL3 non-silent mutation rate between responders and non-responders in the listed cohorts was not significant due to an insufficient sample number, there was some tendency toward a difference. Responders to pembrolizumab and ipilimumab had higher NEIL3 mutation rates in non-small-cell lung cancer and melanoma, respectively. However, in melanoma, responders to pembrolizumab and nivolumab had lower NEIL3 mutation rates (Figure 10F), suggesting that different cancers require personalized customization with regard to immunotherapy.

## 4. Discussion

Currently, the response of patients treated with immunotherapy is diverse among individuals due to the complexity of TME-related factors. Meanwhile, the fact that some population subtypes are not sensitive to immunotherapy should be taken into consideration when administering treatment [46,47]. Due to the need for precision treatment, screening for useful biomarkers to characterize the potential population likely to be responsive to certain therapies is of vital importance. 

Previous studies demonstrated the oncogenic role of NEIL3 in several cancers. However, no comprehensive study has investigated the shared and unique features of NEIL3 in tumors and its relationship with TME. Hence, in this study, we performed a genome-wide, multiomics analysis to assess the pan-cancer characteristics of NEIL3, including expression differences, prognostic value, genetic mutations, related pathways, and immunological features.

Our results revealed that NEIL3 was upregulated in tumor tissues, indicating its wide-range expression across cancers. Combined with prognosis analysis, which showed that higher NEIL3 expression contributes to poorer clinical outcomes, the potential of NEIL3 as an oncogenic predictor in cancer was revealed. Our results are consistent with those of Tran [48], who demonstrated that overexpression of NEIL3 is associated with unfavorable survival in selected types of human cancers. The GSEA results showed that the cell cycle pathway was significantly enriched. In accordance with our results, NEIL3 is known to play an important role in monitoring psoralen interstrand crosslinks by repairing double-strand breaks in human cells [49], an abnormal type of damage induced when cells experience oxidative or inflammatory stress in telomeres during the S/G2 phase [50,51]. Another two enriched pathways were oocyte meiosis and the mTORC1 signaling pathway. Growing evidence has confirmed the function of mTORC1 in the regulation of cell growth, cell survival, and protein synthesis [52,53]. Since upregulated NEIL3 is associated with downregulated mTORC1 signaling, it is reasonable to propose that NEIL3 may have a close relationship with the mTORC1 signaling pathway. Notably, our results also showed that NEIL3 is positively enriched in the intestinal immune network for IgA production. Shinmura K et al. reported that NEIL3 expression was positively correlated with the expression of APOBEC3B, a potent mutation inducer that drives tumor evolution and results in cancer recurrence and metastasis [54]. A study on HCC revealed that APOBEC3B may give rise to the malignant performance of HCC cells by activating intestinal immune networks for IgA production [55]. Taken together, we may reasonably infer that NEIL3 may affect tumorigenesis in coordination with APOBEC3B through the intestinal immune network for IgA production. 

With regard to the immunological features of NEIL3 in TME, our results demonstrated that NEIL3 has multiplex expression patterns associated with neoantigens. Given that neoantigens in different individuals of the same tumor show obvious individual heterogeneity [56], patients with different NEIL3 expression in various cancers should be categorized when considering neoantigen-specific therapy. In our study, NEIL3 expression was related to an elevated level of TMB in a majority of cancer types. In accordance with our previous result, NEIL3 presented a high mutation frequency among cancers. NEIL3 can easily mutate and, consequently, increases the number of neoepitopes, which are more likely to be neoantigens in some cancers. However, certain tumors may achieve evolution through frequent mutation of NEIL3. More studies are warranted to explore this issue. Currently, FDA approval has been obtained for the use of high MSI as a marker for the selection of patients for ICI treatment [57]. Our analysis illustrated that NEIL3 expression is associated with MSI in several cancers, such as STAD, SARC, and DLBC. However, NEIL3 expression does not synchronize with the MSI level, suggesting that it may be an independent novel biomarker in therapy administration. Additionally, evaluation of the association between NEIL3 and MMR genes further confirmed its oncogenic role as a DNA damage repair enzyme in cancers. According to a previous study [58], NEIL3 elevated the expression of EMT factors, including the E/N-cadherin switch and the transcription of MMP genes, and promoted the invasion, migration, and stemness phenotypes in hepatocellular carcinoma cells. Moreover, they found that NEIL3 directly interacted with the key EMT player, TWIST1, enhancing its binding to the canonical E-box promoter region, which did not require its enzymatic activity. Notably, higher expression of NEIL3 is positively correlated with higher EMT activity in BLCA, LIHC, and THCA, which implied the potential reason for the poorer prognosis and metastasis of these three kinds of cancers to some extent. 

As is known, the TME characteristic can be broadly categorized as inflamed or non-inflamed [59,60]. Tumors with a high density of tumor-infiltrating immunocytes may be immunologically sensitive to specific immunotherapy. Current clinical data suggested that ICIs mostly function by reinvigorating preexisting antitumor T-cell responses and are most effective in inflamed tumors [61]. Based on the results of immunocytes infiltration, TMB, and immune subtypes, we preliminarily presented the potential impact of NEIL3 expression on TME and the phenotypic changes in selected cancers. Generally, NEIL3 expression is closely associated with the C1 (wound healing) and C2 (IFNγ-dominant) immune subtypes in a majority of cancers. In our study, NEIL3 is positively correlated with six types of immune cell infiltration, including B cells, CD4 T cells, CD8 T cells, neutrophils, macrophages, DCs in KIPAN, KIRC, and LIHC, indicating that these three types of cancers may be inflamed tumors. Previously, Teng et al. mimicked the renal cell carcinoma (RCC) microenvironment to investigate its effect on DCs. The results showed that the isolated conditional media (CM) from RCC can inhibit the maturation of DCs and impair their function, and DCs treated with RCC CM induced the increase of Treg cells, which may account for the RCC escaping from anti-tumor immunity [62]. In LIHC, we identified that high expression of NEIL3 was also positively associated with high infiltration of DCs. DCs are innate immune cells that play a crucial role in anti-tumor immunity. Nevertheless, a recent study found that the enriched Treg cells in HCC can interact with type 2 conventional DCs, inducing the loss of antigen-presenting HLA-DR, and shaping the immunosuppressive TME, thus resulting in the immune evasion of HCC [63]. Taken together, these studies indicated that with upregulated NEIL3 expression, RCC and HCC may activate the tumor-suppressive function of the infiltrated immunocytes through cell interplay in TME, despite having substantial immune components.

Next, we discovered that NEIL3 expression was strongly positively correlated with DNA methyltransferase gene expression in most cancers. Olsen et al. reported that NEIL3 was correlated with DNA-methylationed genes in proliferation and myofibroblast differentiation pathways in a myocardial infarction mouse model [64], which verified our results. DNA methyltransferase can epigenetically silence immune-protective genes, as in cancer, and lead to immunosuppression [65]. 

With the widespread utilization of ICIs, undesirable effects appear unexpectedly, one of which is the low response rate of anti-PD-1/PD-L1 therapy [66]. This consequence may be attributed to individual heterogeneity. Our study revealed that different NEIL3 expression levels and non-silent mutation rates influence the ICI response to some extent, which relies on cancer and ICI types. As demonstrated by our results, responsive patients with urothelial cancer undergoing atezolizumab treatment possess significantly higher expression levels of NEIL3 than unresponsive patients. Regardless of the satisfactory tolerance, approximately 9% of atezolizumab-treated patients with advanced urothelial cancer showed grade 3–4 toxic events in a phase-I clinical trial [67]. Therefore, the significance of dividing patients into different therapeutic subgroups based on reliable parameters cannot be neglected. Our study suggests that urothelial cancer cells expressing high NEIL3 levels are more likely to respond to and benefit from atezolizumab. The smoking subgroup in conjunction with high NEIL3 expression in urothelial cancer may also be more responsive to atezolizumab; however, due to the small sample size, this hypothesis should be further confirmed in future studies. Additionally, in our study, responsive melanoma patients treated with ipilimumab presented a higher non-silent mutation rate of NEIL3 than non-responsive patients. Melanoma is a type of cancer particularly sensitive to immunotherapy. Previous studies demonstrated that patients with a higher somatic tumor mutation benefited more from ICI [46]. Although without statistical significance, our results suggested the possibility that ipilimumab may demonstrate greater efficacy in melanoma patients with a high non-silent mutation rate of NEIL3. Hence, the expression and mutation level of NEIL3 may be a promising biomarker to predict the efficacy of ICI treatments. 

The combined application of chemotherapy and immunotherapy has gradually become a current trend in cancer treatment. The monotherapy of the two has proven to be effective in increasing response and ameliorating prognosis among several cancers [68,69]. Thus, we investigated the association between NEIL3 expression and chemotherapeutic drug efficacy. In general, we observed a positive correlation between NEIL3 expression and the drug efficacy of chemotherapeutic drugs, implying the great potential of NEIL3 as an indicator of chemotherapy efficacy. Sorafenib has been the first-line drug commonly used in late-stage HCC. Our results showed that high NEIL3 expression is related to better efficacy of sorafenib, suggesting that NEIL3 expression levels may be a promising tool with which to identify potential patients who may benefit from sorafenib treatment. In addition, a recent study discovered that the loss of NEIL3 promotes chemotherapy resistance in prostate cancer, further proving our result from the opposite side [12]. Taken together, our results reflected that NEIL3 has an important value in therapeutic prediction and may function as a sensitive indicator in chemotherapy.

With several strengths, the results of this study are reasonable. First, this study included data from multiple databases, which enhances the reliability of our conclusions. Second, we performed experiments to validate the results of bioinformatics analysis, the results of which are aligned. Moreover, we applied a pooled assessment of sequencing data from several clinical trials, enabling us to gain insight into the possible utility of NEIL3 in practical applications. There are some limitations of our study. First, we lack causative data showing the effects of NEIL3 knockout in culture systems or preclinical model prognosis, which warrants further exploration. Second, several of the included clinical trials had small sample sizes, which may obscure the true relationship between NEIL3 expression and immunotherapeutic regimens. Despite these limitations, the virtues of this study should be weighed against the limitations. To the best of our knowledge, this is the first study to report the immunological features of NEIL3 among various cancers. Our research demonstrated that NEIL3 may be an indicator for both prognosis prediction and drug stratification, and it highlights the promising effect of NEIL3 in tumor therapy, which provides a foundation for future studies.

## 5. Conclusions

This study revealed that NEIL3 has a strong association with the immune microenvironment and phenotypic changes in selected types of cancers, such as KIRC, LIHC, and LUAD. NEIL3 may be utilized as a prognostic biomarker and provides insight into the efficacy of chemotherapy and immunotherapy.

## Figures and Tables

**Table 1:** The prediction of functional changes in NEIL3 with certain site mutations.

Position	AA1	AA2	Result	Score	Sensitivity	Specificity
76	V	L	Possibly Damaging	0.604	0.87	0.91
96	R	W	Possibly Damaging	0.918	0.81	0.94
103	M	I	Benign	0.000	1.00	0.00
106	P	L	Benign	0.102	0.93	0.85
132	D	H	Probably Damaging	0.998	0.27	0.99
225	I	M	Possibly Damaging	0.950	0.79	0.95
234	R	M	Probably Damaging	0.986	0.74	0.96
247	K	T	Probably Damaging	1.000	0.00	1.00
344	R	K	Benign	0.145	0.92	0.86
390	L	V	Probably Damaging	0.999	0.14	0.99
481	N	D	Benign	0.097	0.93	0.85
498	R	H	Benign	0.000	1.00	0.00
559	R	C	Probably Damaging	1.000	0.00	1.00
559	R	H	Probably Damaging	1.000	0.00	1.00

Note: AA1, AA2: amino acid allele; AA1-AA2 refers to single-nucleotide polymorphism (SNP) mutations from AA1 to AA2.

## Data Availability

All data used in this study were publicly available prior to analysis (Methods).

## Supplementary Materials

The following supporting information can be downloaded at: https://www.mdpi.com/article/10.3390/cancers15010109/s1, Figure S1: NEIL3 may function as a prognostic factor in LIHC; Figure S2: NEIL3 expression is correlated with high tumor stage; Figure S3: NEIL3 expression is correlated with high tumor grade; Figure S4: Relationship of NEIL3 expression with patient DFI; Figure S5: The correlation of NEIL3 expression and DSS; Figure S6: The correlation of NEIL3 expression and patient OS; Figure S7: The correlation of NEIL3 expression and patient PFI; Figure S8: Correlation of NEIL3 mutation and immune checkpoint genes; Figure S9: Correlation of NEIL3 expression and immunocyte infiltration in 33 types of cancers; Figure S10: Relationship between NEIL3 expression and immune Score in 33 types of cancers; Figure S11: Relationship between NEIL3 expression and ESTIMATE score in 33 types of cancers; Figure S12: Relationship between NEIL3 expression and stromal score in 33 types of cancers; Figure S13: NEIL3 expression is related to multiple immune biomarkers across cancers; Figure S14: NEIL3 expression is related to different immune subtypes across cancers; C1, red; C2, yellow; C3, green; C4, blue; C5, purple; C6, orange; Figure S15: Activity of EMT pathway between high and low NEIL3 expression groups in pan-cancer; Figure S16: Signaling pathway enrichment with NEIL3 expression; Figure S17: Orignal Western Blots; Table S1: Primers for RT-qPCR; Table S2: Clinical characteristics of HCC patients.

## Abbreviations

ACC: adrenocortical carcinoma; BLCA, bladder urothelial carcinoma; CAN, copy number alteration; CARE, Computational Analysis of Resistance; CCLE, Cancer Cell Line Encyclopedia; CGP, Cancer Genome Project; COADREAD, colon adenocarcinoma/rectum adenocarcinoma esophageal carcinoma; CTLA-4, cytotoxic T-lymphocyte associated protein 4; CTRP, Cancer Therapeutics Response Portal; DCs, dendritic cells; DFI, disease-free interval; DSS, disease-specific survival; GBM, glioblastoma multiforme; GEPIA2, Gene Expression Profiling Interactive Analysis 2; GSEA, Gene Set Enrichment Analysis; GSVA, Gene Set Variation Analysis; GTEx, Genotype-Tissue Expression; HCC, hepatocellular carcinoma; HNSC, head and neck squamous cell carcinoma; ICI, immune checkpoint inhibitor; KEGG, Kyoto Encyclopedia of Genes and Genomes; KICH, kidney chromophobe; KIPAN, pan-kidney cancer cohort; KIRC, kidney renal clear cell carcinoma; KIRP, kidney renal papillary cell carcinoma; LGG, lower-grade glioma; LIHC, liver hepatocellular carcinoma; LUAD, lung adenocarcinoma; LUSC, lung squamous cell carcinoma; MHCII, MHC class II; MMR, mismatch repair; MSI, microsatellite instability; NEIL3, Endonuclease VIII-like protein 3; NGS, next-generation sequencing; OS, overall survival; PAAD, pancreatic adenocarcinoma; PD-1, programmed cell death protein 1; PFI, progression-free interval; PRAD, prostate adenocarcinoma; RCC, renal cell carcinoma; SARC, sarcoma; STAD, stomach adenocarcinoma; STES, stomach and esophageal carcinoma; TCGA, The Cancer Genome Atlas; TGCT, testicular germ cell tumor; THCA, thyroid carcinoma; THYM, thymoma; TIMER, Tumor Immune Estimation Resource database; TISIDB, Tumor-immune System Interactions Database; TMB, tumor mutational burden; TME, tumor microenvironment; UCEC, uterine corpus endometrial carcinoma; zf-GRF, GRF zinc finger; Zf-R, Zn-finger in Ran binding protein.
