# Supplementary material for: Pan-Cancer Landscape of NEIL3 in Tumor Microenvironment: A Promising Predictor for Chemotherapy and Immunotherapy
Source: Cancers (Basel). 2022 Dec 24;15(1):109. doi: 10.3390/cancers15010109 (PMC9817722; doi:10.3390/cancers15010109)
Supplement: Supplementary file 1 [file cancers-15-00109-s001.zip › Supplementary Fig S16 GSEA.pdf]

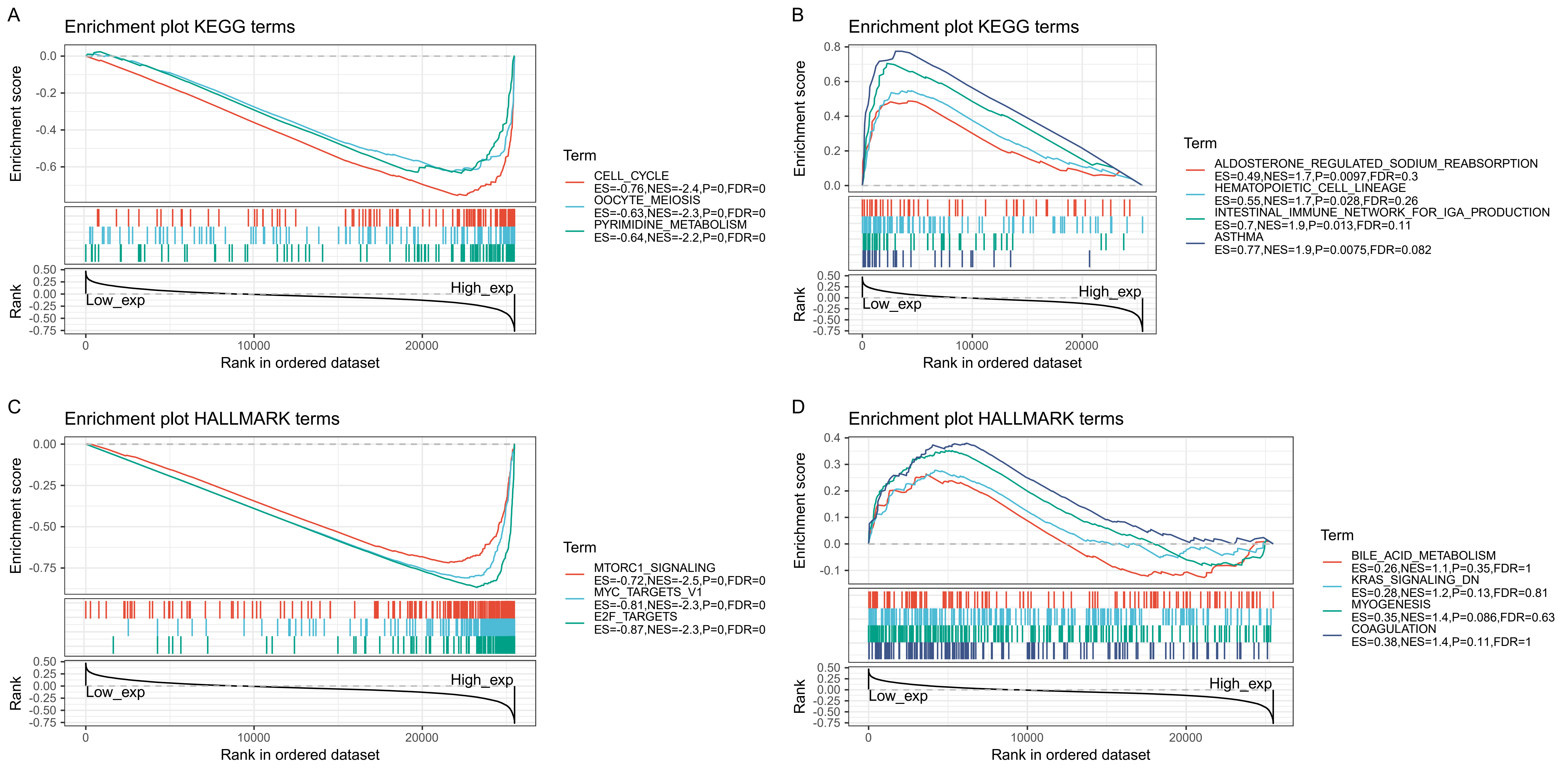

A
Enrichment plot KEGG terms
Enrichment score
Rank
Low_exp
High_exp
Rank in ordered dataset
Term
CELL_CYCLE
ES=-0.76,NES=-2.4,P=0,FDR=0
OOCYTE_MEIOSIS
ES=-0.63,NES=-2.3,P=0,FDR=0
PYRIMIDINE_METABOLISM
ES=-0.64,NES=-2.2,P=0,FDR=0
B
Enrichment plot KEGG terms
Enrichment score
Rank
Low_exp
High_exp
Rank in ordered dataset
Term
ALDOSTERONE_REGULATED_SODIUM_REABSORPTION
ES=0.49,NES=1.7,P=0.0097,FDR=0.3
HEMATOPOIETIC_CELL_LINEAGE
ES=0.55,NES=1.7,P=0.028,FDR=0.26
INTESTINAL_IMMUNE_NETWORK_FOR_IGA_PRODUCTION
ES=0.7,NES=1.9,P=0.013,FDR=0.11
ASTHMA
ES=0.77,NES=1.9,P=0.0075,FDR=0.082
C
Enrichment plot HALLMARK terms
Enrichment score
Rank
Low_exp
High_exp
Rank in ordered dataset
Term
MTORC1_SIGNALING
ES=-0.72,NES=-2.5,P=0,FDR=0
MYC_TARGETS_V1
ES=-0.81,NES=-2.3,P=0,FDR=0
E2F_TARGETS
ES=-0.87,NES=-2.3,P=0,FDR=0
D
Enrichment plot HALLMARK terms
Enrichment score
Rank
Low_exp
High_exp
Rank in ordered dataset
Term
BILE_ACID_METABOLISM
ES=0.26,NES=1.1,P=0.35,FDR=1
KRAS_SIGNALING_DN
ES=0.28,NES=1.2,P=0.13,FDR=0.81
MYOGENESIS
ES=0.35,NES=1.4,P=0.086,FDR=0.63
COAGULATION
ES=0.38,NES=1.4,P=0.11,FDR=1
