# Supplementary material for: Pan-Cancer Landscape of NEIL3 in Tumor Microenvironment: A Promising Predictor for Chemotherapy and Immunotherapy
Source: Cancers (Basel). 2022 Dec 24;15(1):109. doi: 10.3390/cancers15010109 (PMC9817722; doi:10.3390/cancers15010109)
Supplement: Supplementary file 1 [file cancers-15-00109-s001.zip › Supplementary Fig S2 grade.pdf]

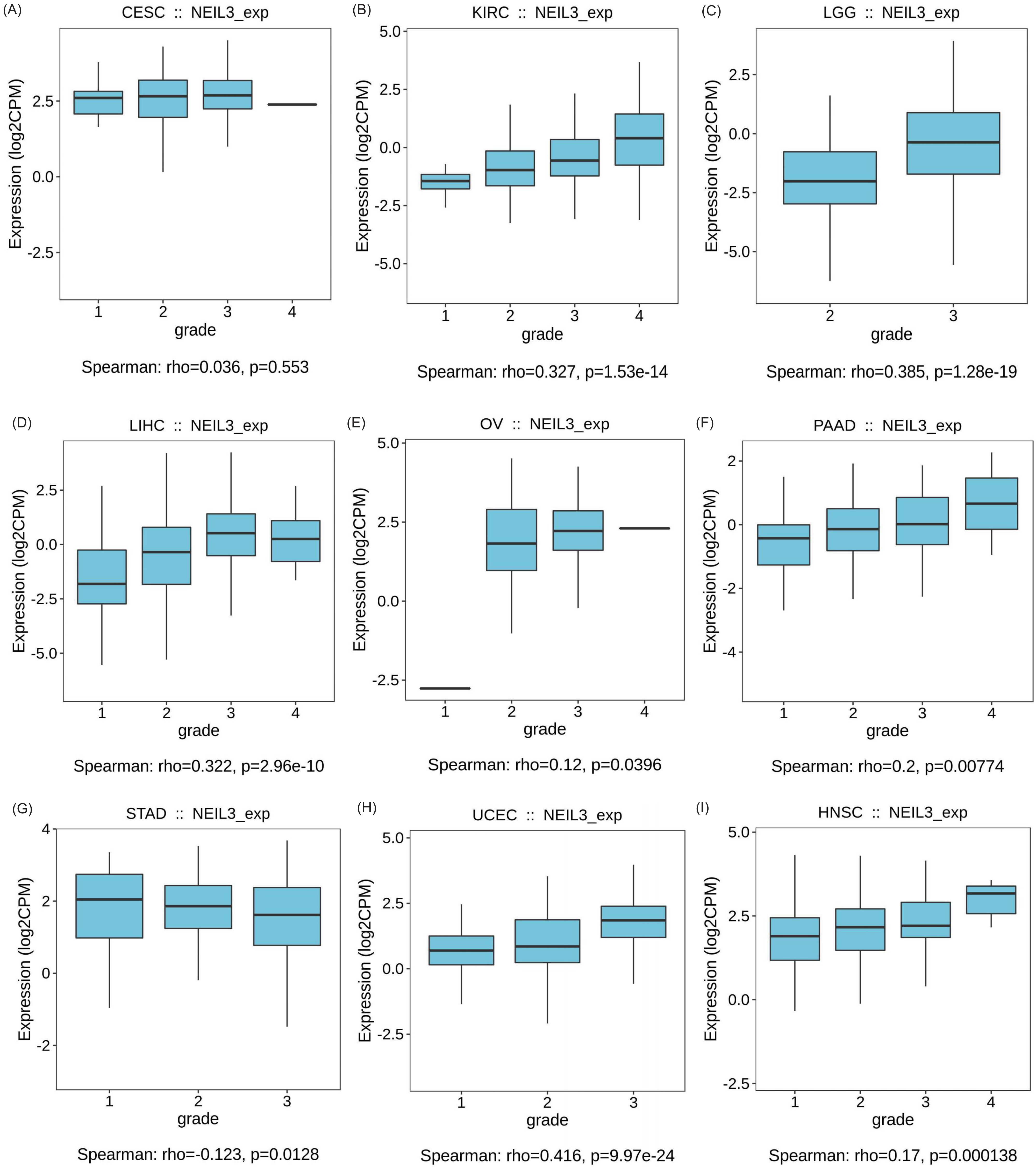
(A)
CESC :: NEIL3_exp
Expression (log2CPM)
grade
Spearman: rho=0.036, p=0.553
(B)
KIRC :: NEIL3_exp
Expression (log2CPM)
grade
Spearman: rho=0.327, p=1.53e-14
(C)
LGG :: NEIL3_exp
Expression (log2CPM)
grade
Spearman: rho=0.385, p=1.28e-19
(D)
LIHC :: NEIL3_exp
Expression (log2CPM)
grade
Spearman: rho=0.322, p=2.96e-10
(E)
OV :: NEIL3_exp
Expression (log2CPM)
grade
Spearman: rho=0.12, p=0.0396
(F)
PAAD :: NEIL3_exp
Expression (log2CPM)
grade
Spearman: rho=0.2, p=0.00774
(G)
STAD :: NEIL3_exp
Expression (log2CPM)
grade
Spearman: rho=-0.123, p=0.0128
(H)
UCEC :: NEIL3_exp
Expression (log2CPM)
grade
Spearman: rho=0.416, p=9.97e-24
(I)
HNSC :: NEIL3_exp
Expression (log2CPM)
grade
Spearman: rho=0.17, p=0.000138
