# Supplementary figures and images for: Pan-Cancer Landscape of NEIL3 in Tumor Microenvironment: A Promising Predictor for Chemotherapy and Immunotherapy
Source: Cancers (Basel). 2022 Dec 24;15(1):109. doi: 10.3390/cancers15010109 (PMC9817722; doi:10.3390/cancers15010109)

A

Normal

Tumor

NEIL3

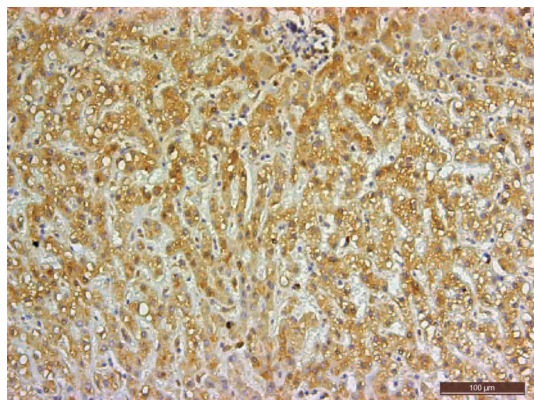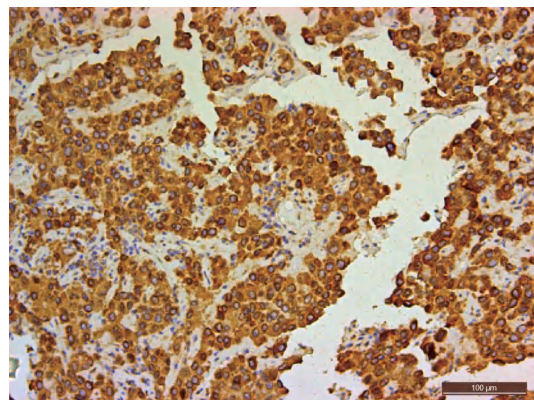

B

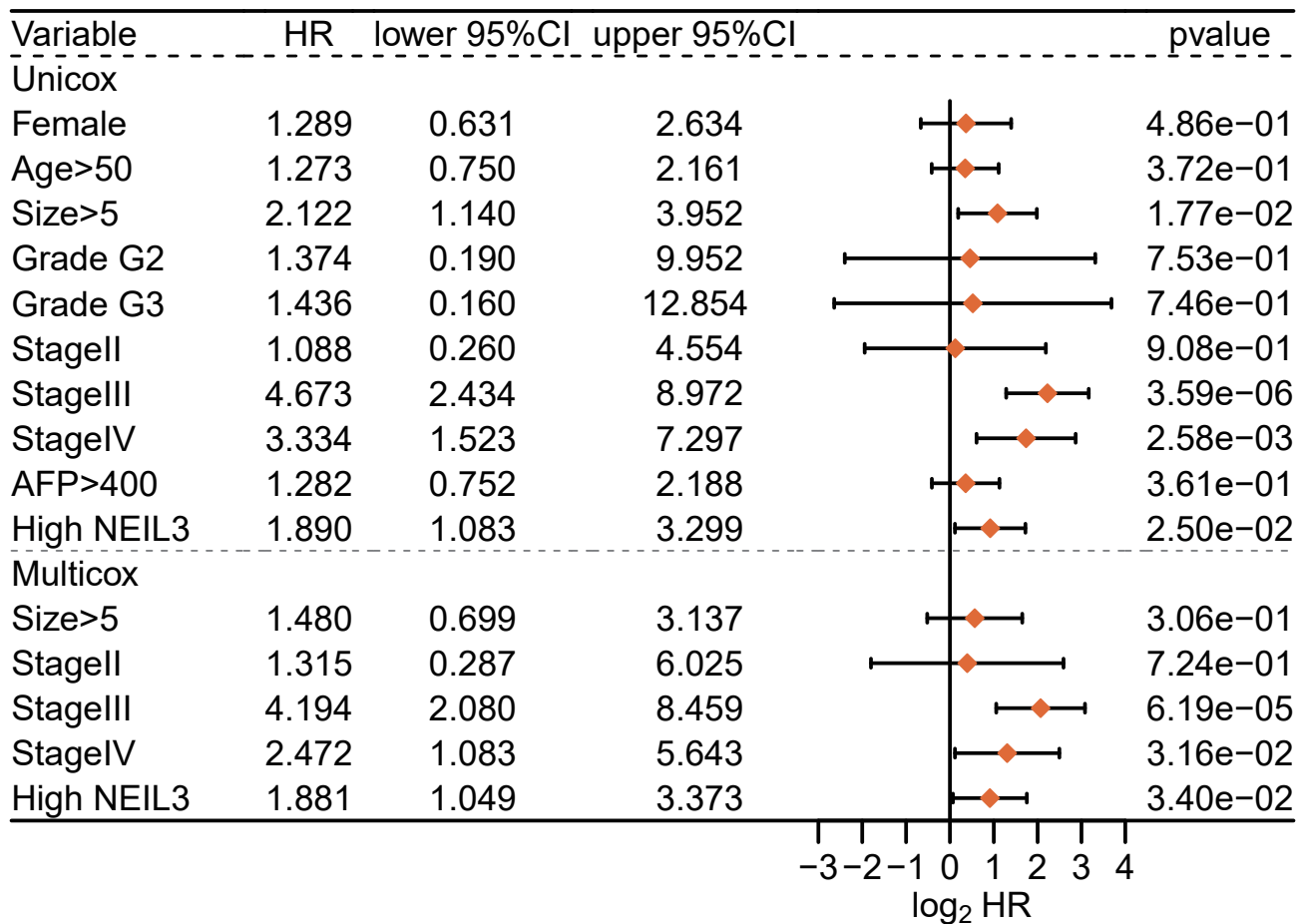

Supplement: Supplementary file 1 [file cancers-15-00109-s001.zip › Supplementary Fig S1 forest.pdf]

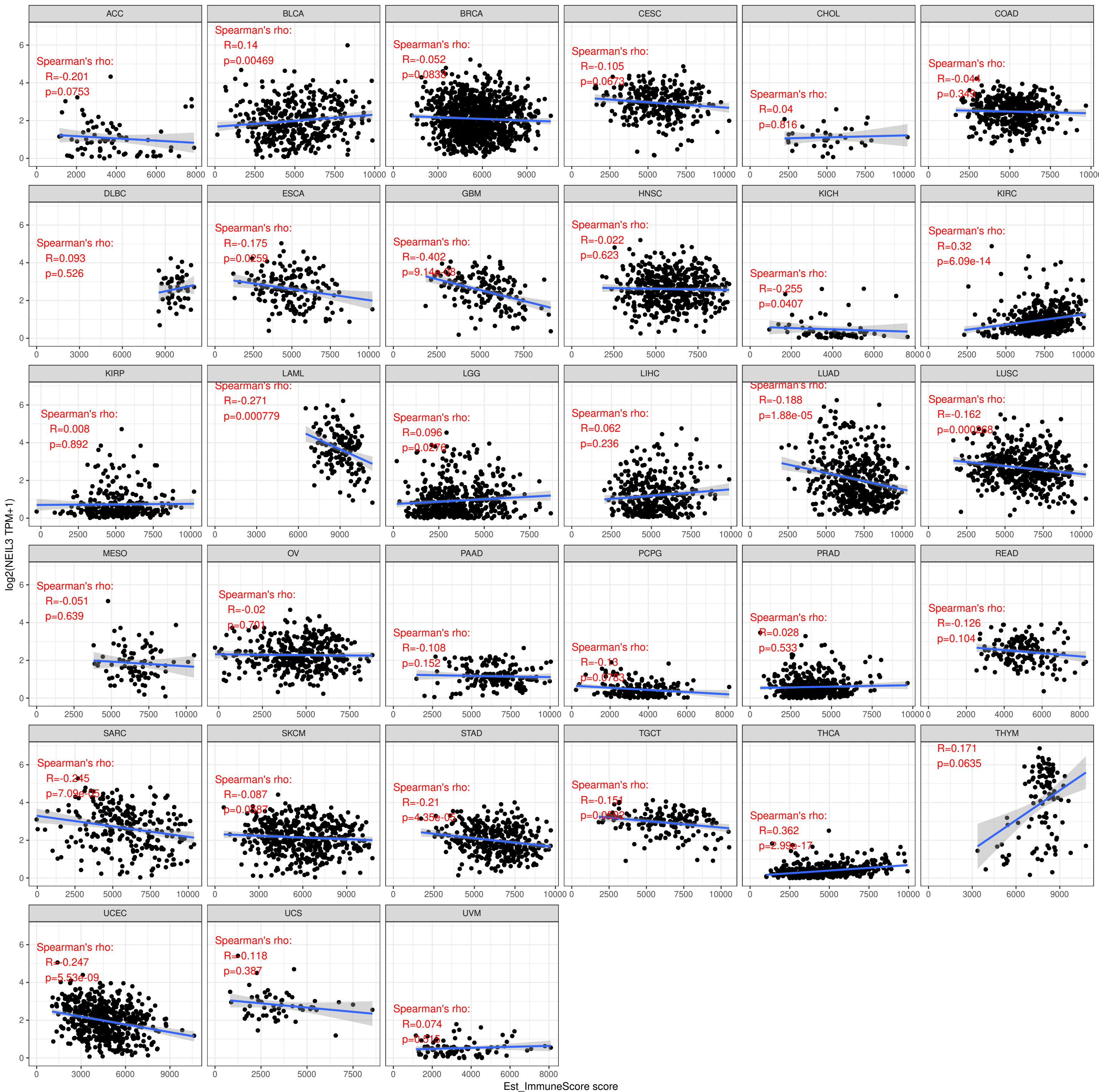

Supplement: Supplementary file 1 [file cancers-15-00109-s001.zip › Supplementary Fig S10 Est Immune Score.pdf]

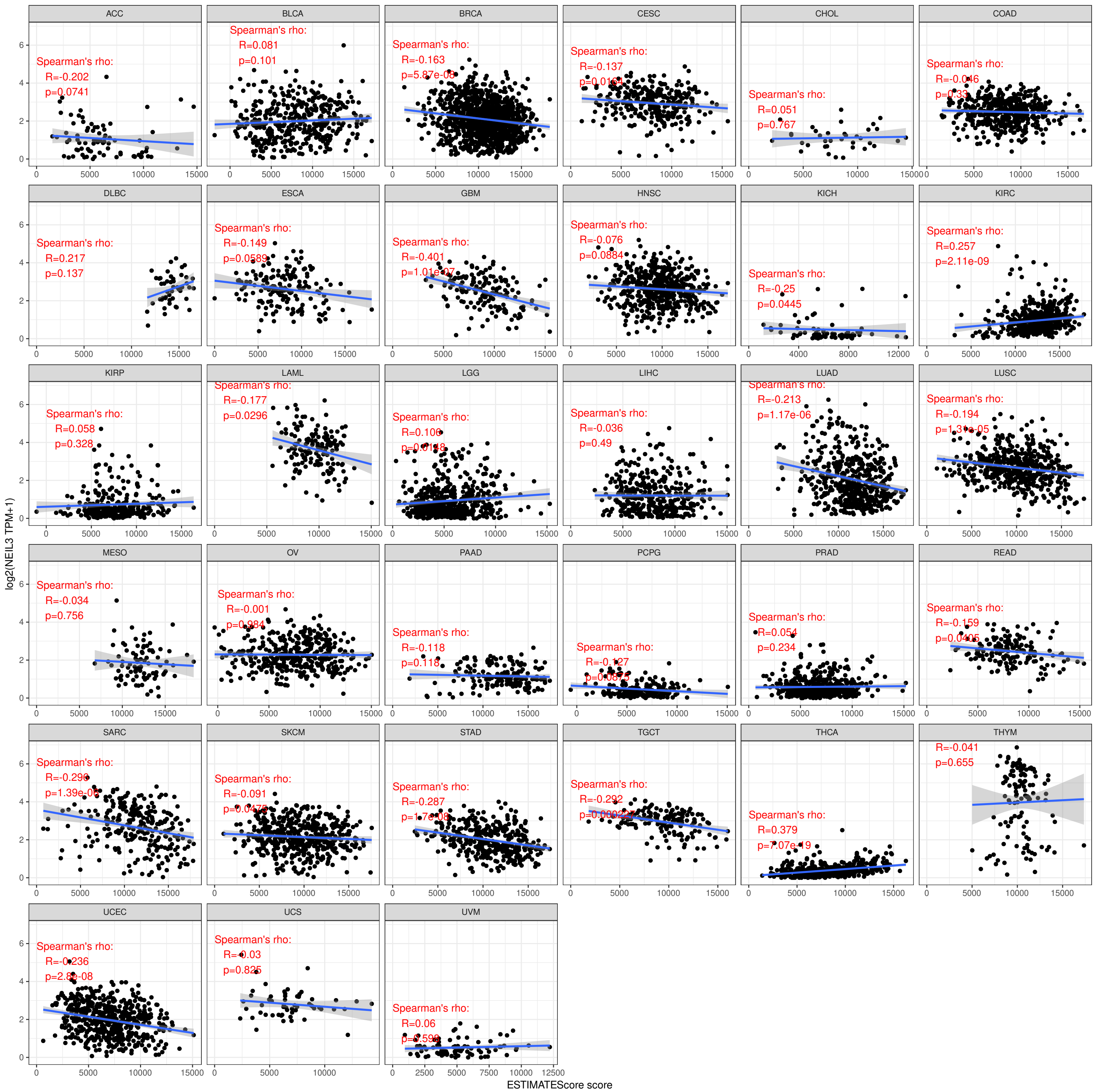

Supplement: Supplementary file 1 [file cancers-15-00109-s001.zip › Supplementary Fig S11 ESTIMATE Score.pdf]

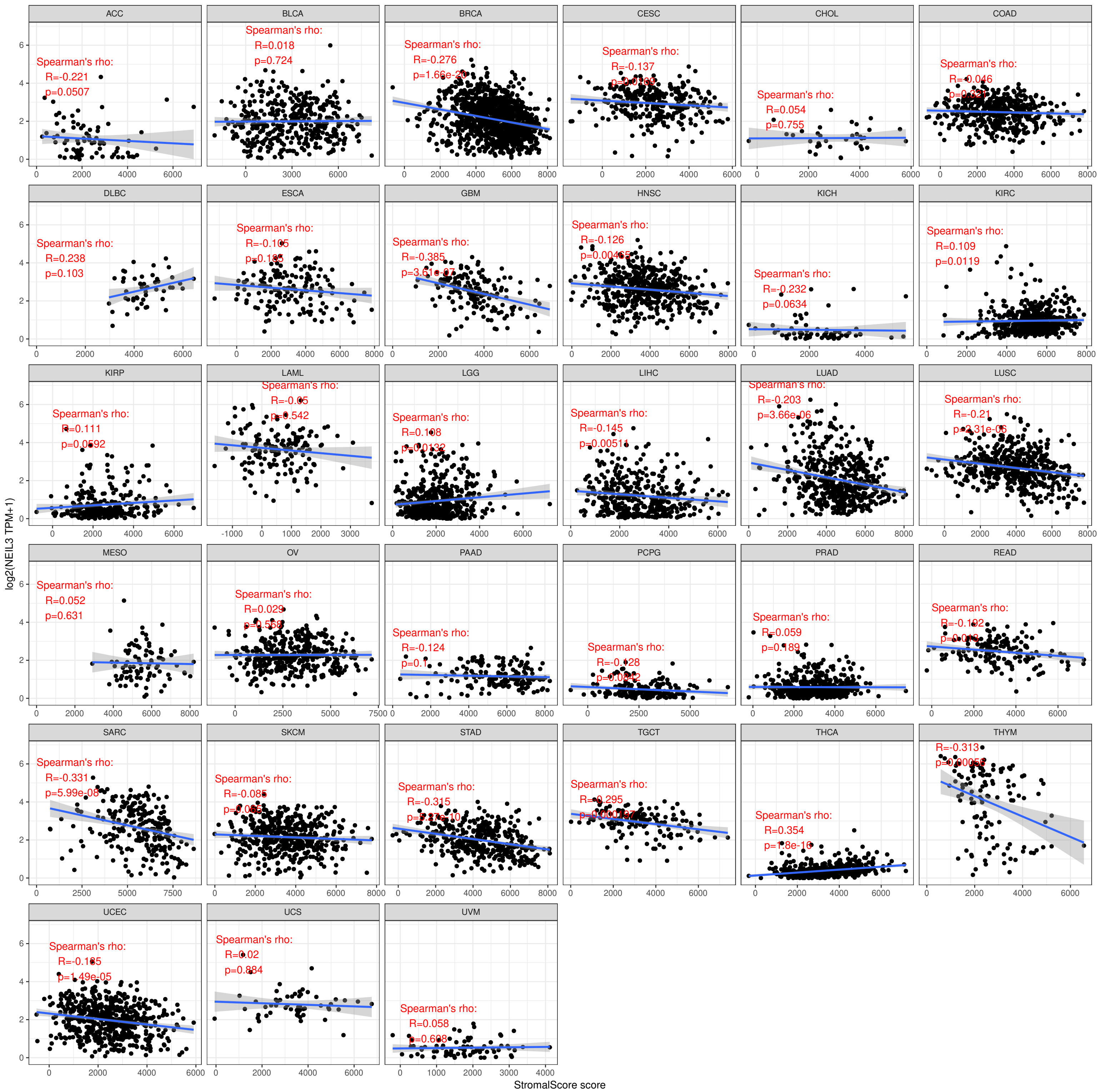

Supplement: Supplementary file 1 [file cancers-15-00109-s001.zip › Supplementary Fig S12 Stromal Score.pdf]

**A**

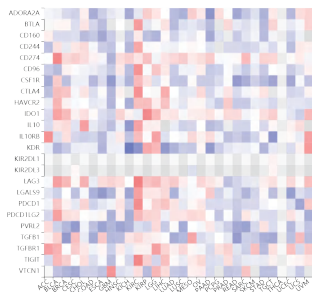

**B**

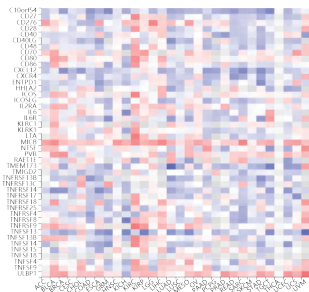

**C**

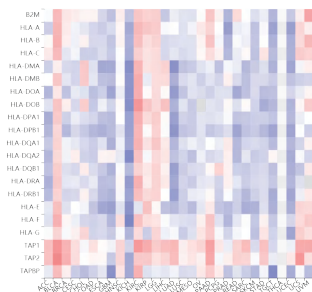

## D

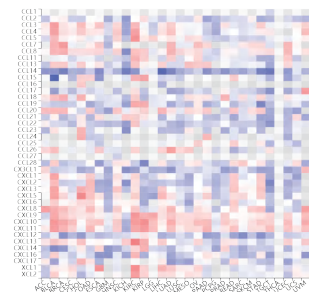

## E

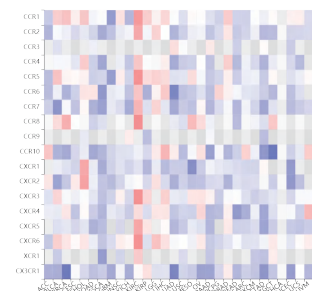

**F**

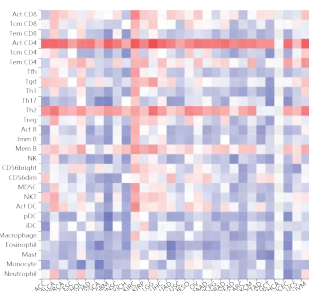

Supplement: Supplementary file 1 [file cancers-15-00109-s001.zip › Supplementary Fig S13 inflammatory factor.pdf]

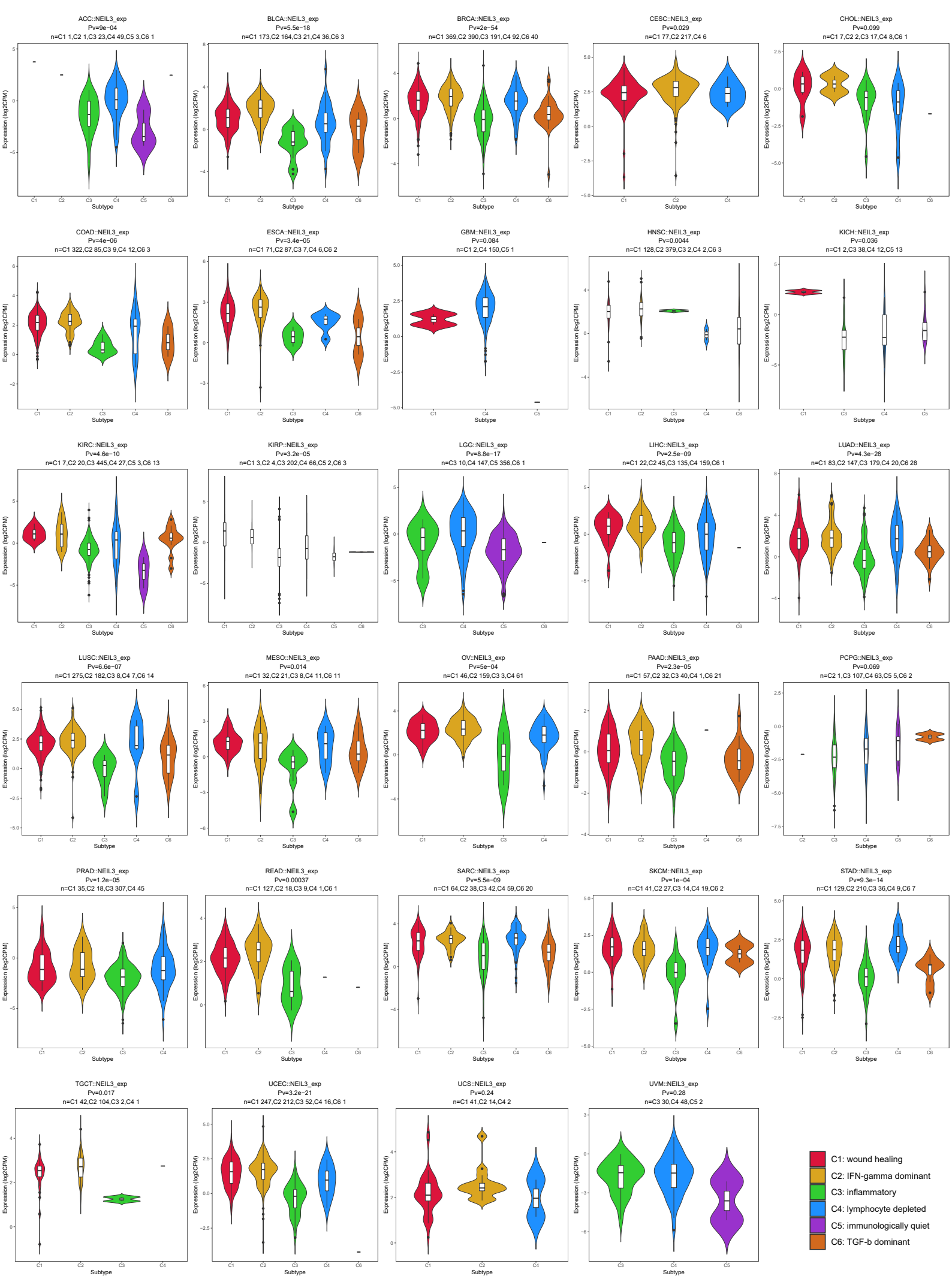

Supplement: Supplementary file 1 [file cancers-15-00109-s001.zip › Supplementary Fig S14 immune subtypes.pdf]

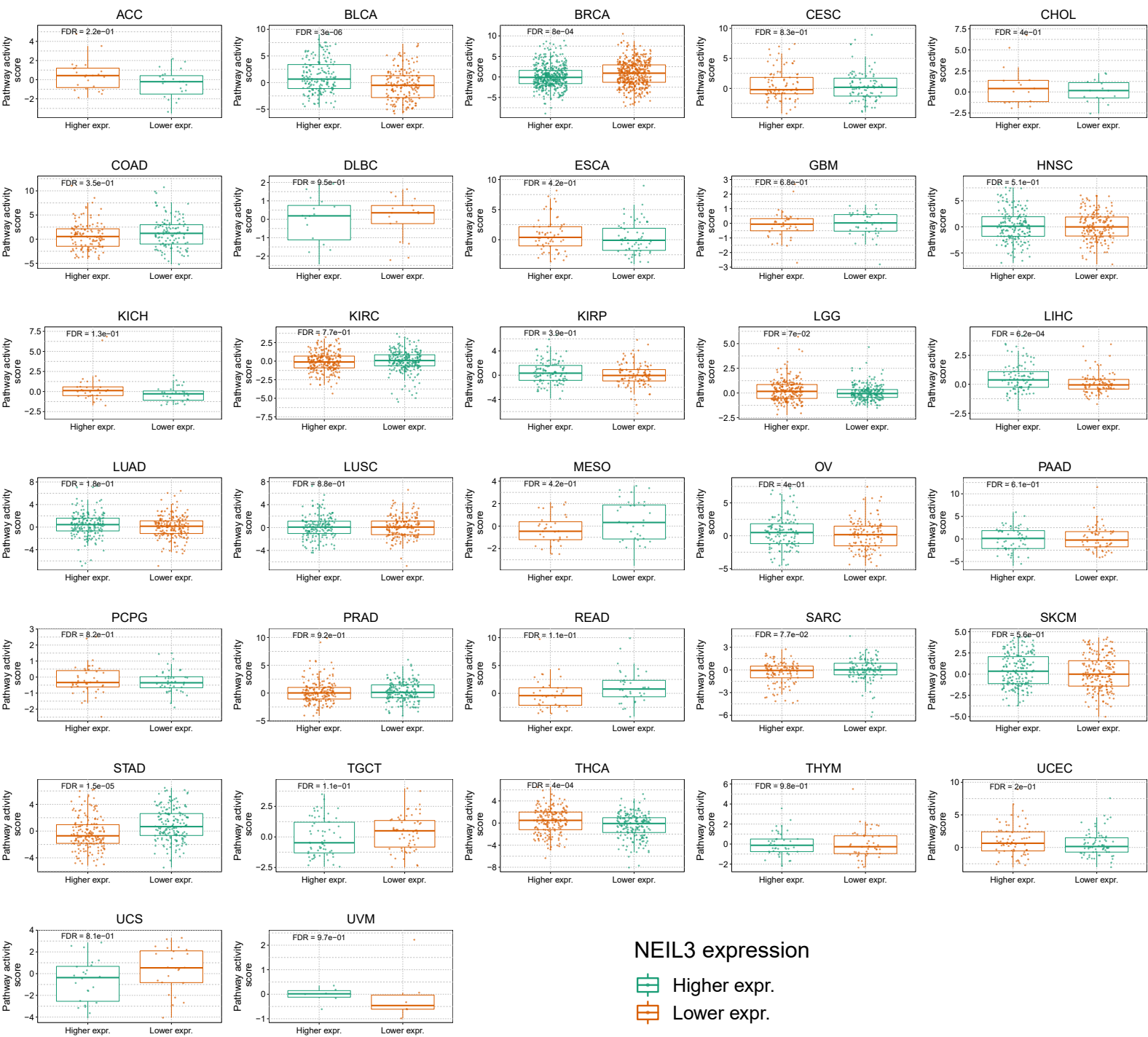

NEIL3 expression

Higher expr.

Lower expr.

Supplement: Supplementary file 1 [file cancers-15-00109-s001.zip › Supplementary Fig S15 EMT.pdf]

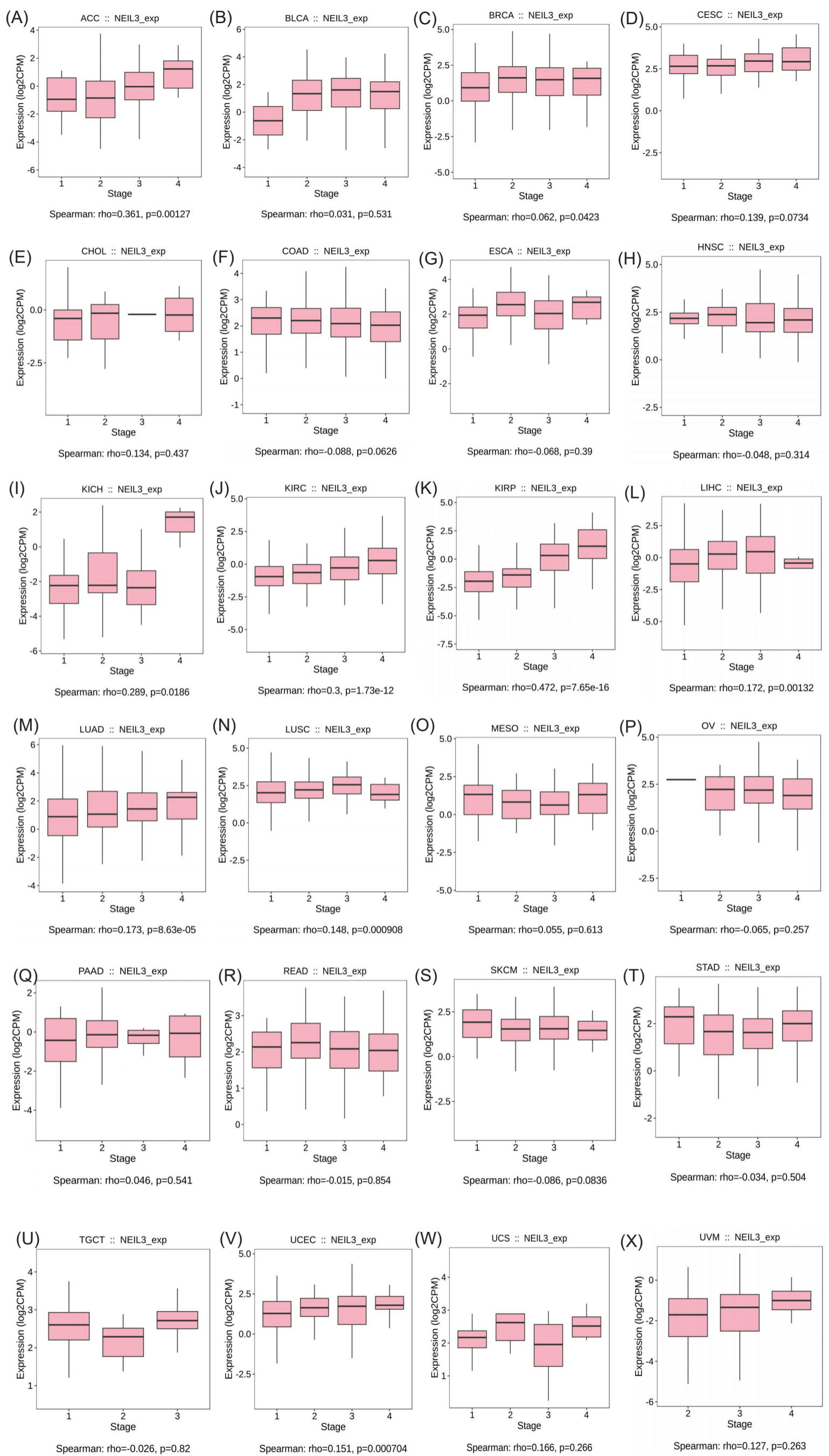

Supplement: Supplementary file 1 [file cancers-15-00109-s001.zip › Supplementary Fig S3 stage.pdf]

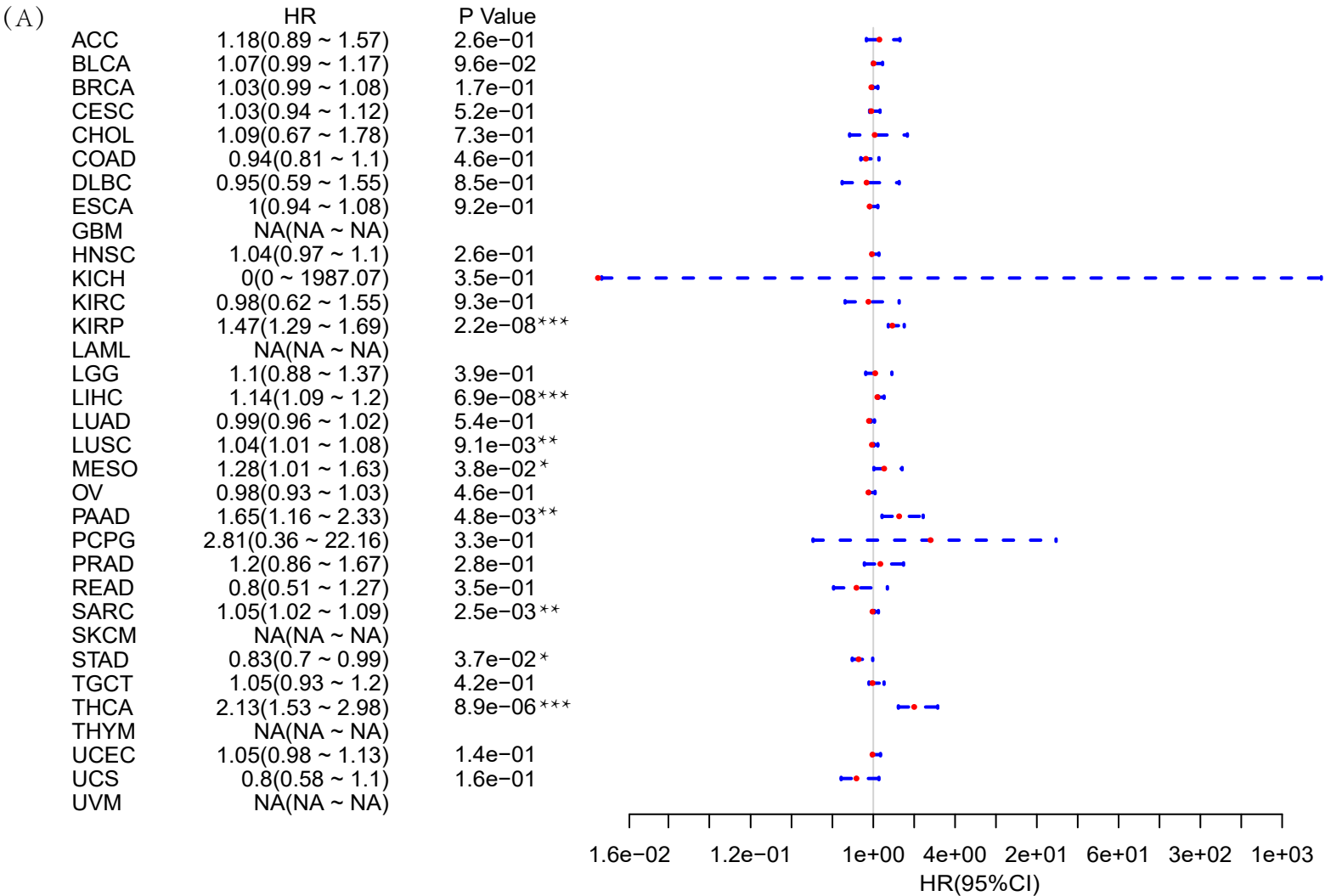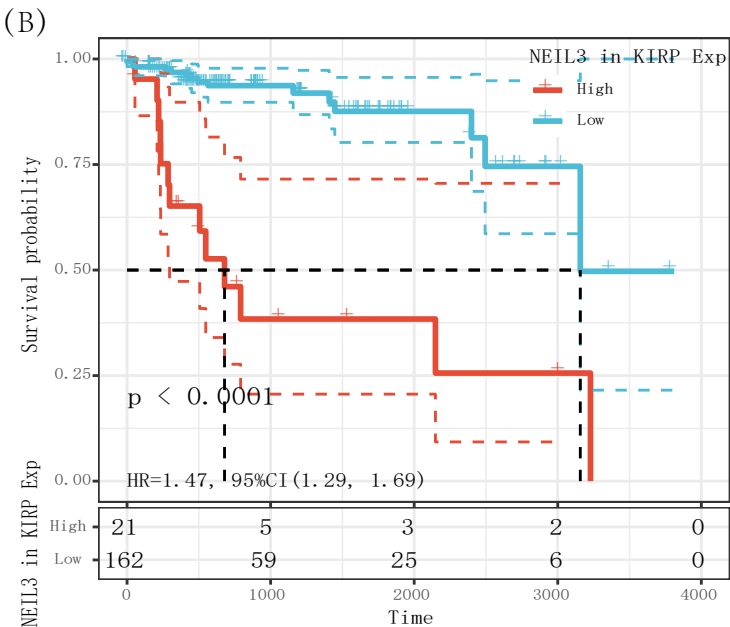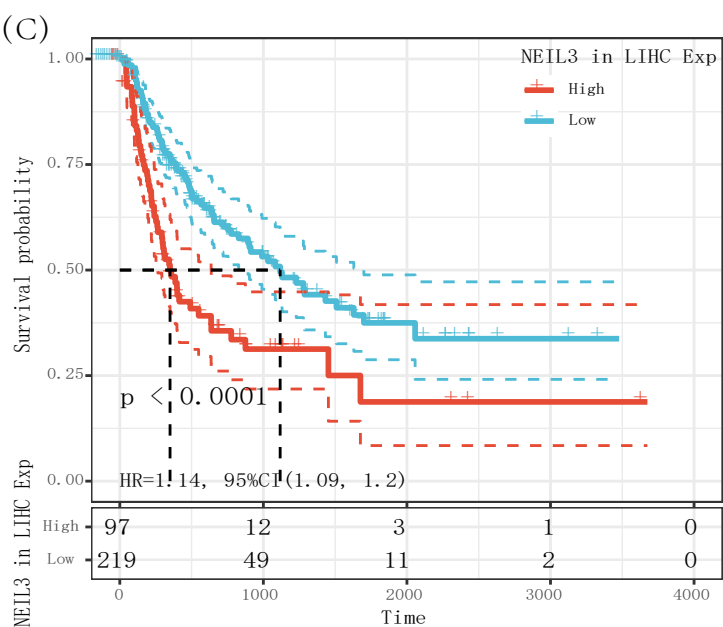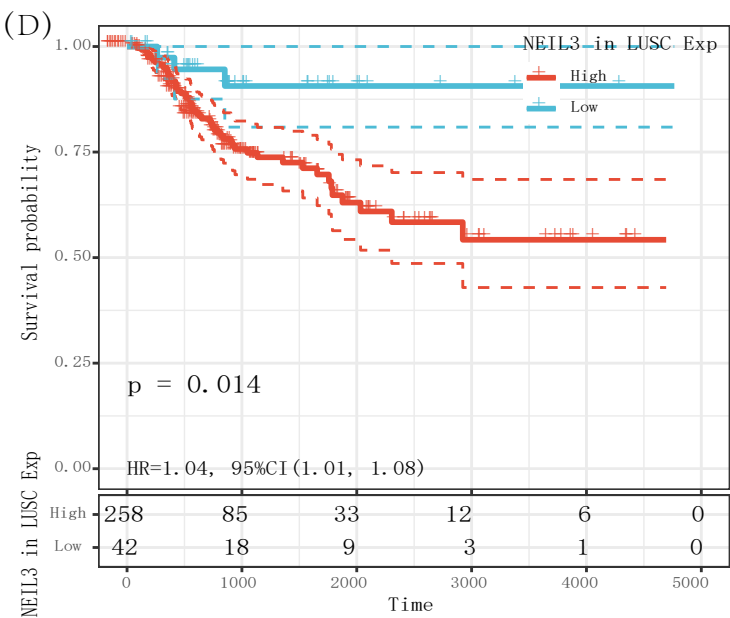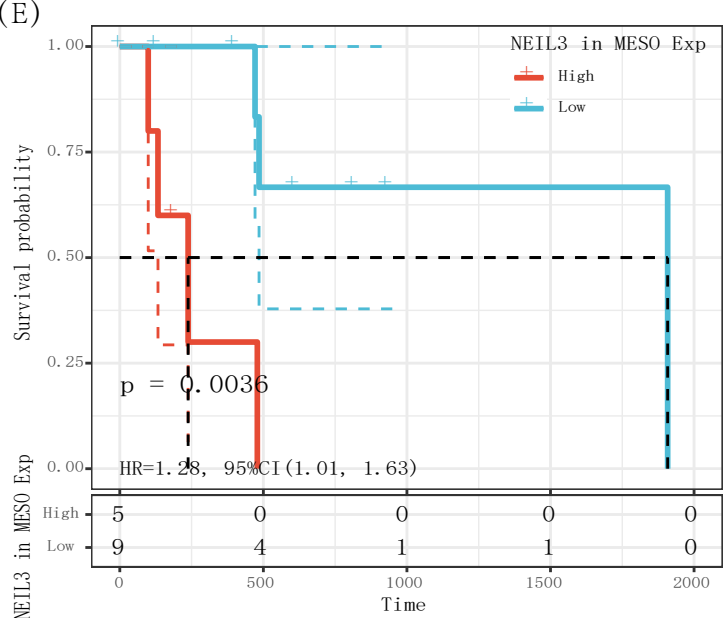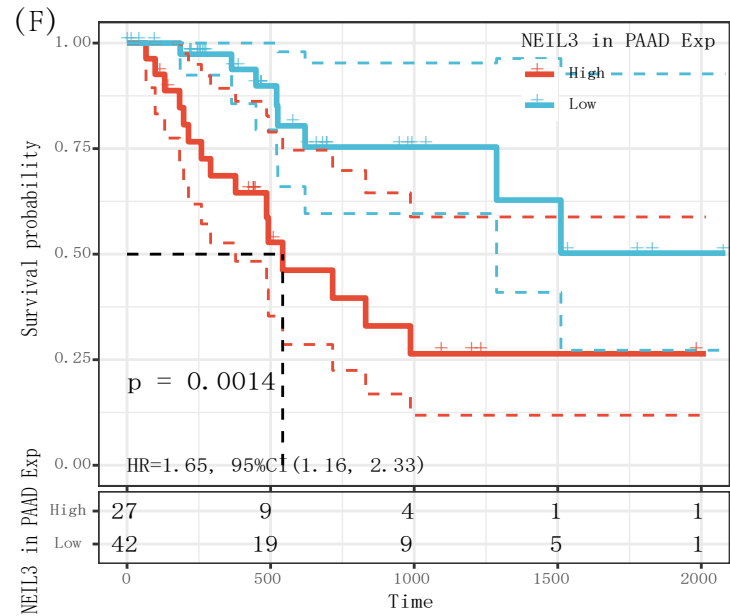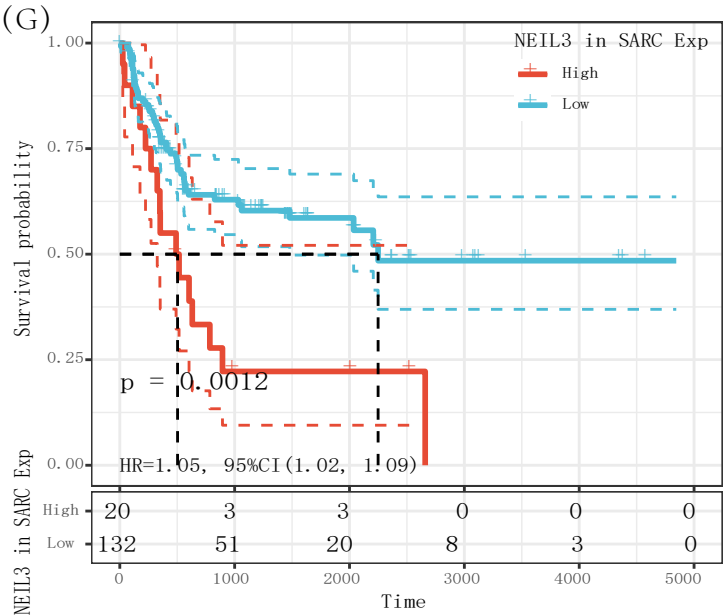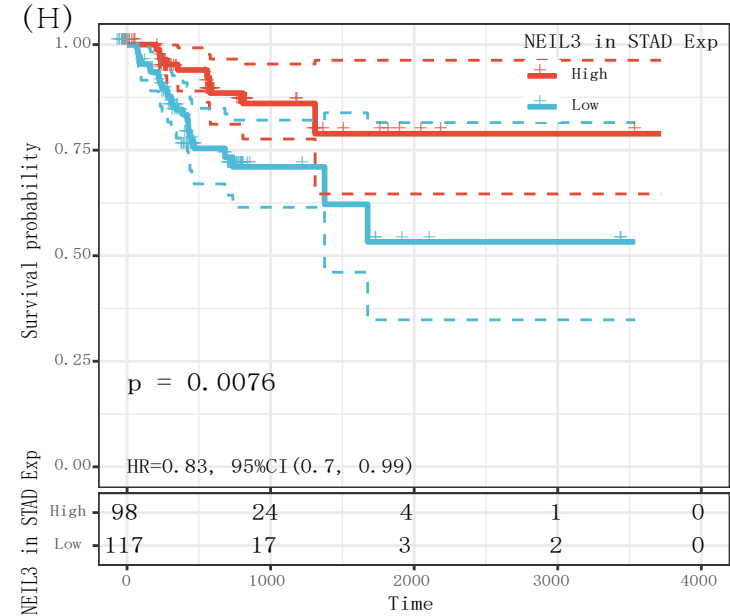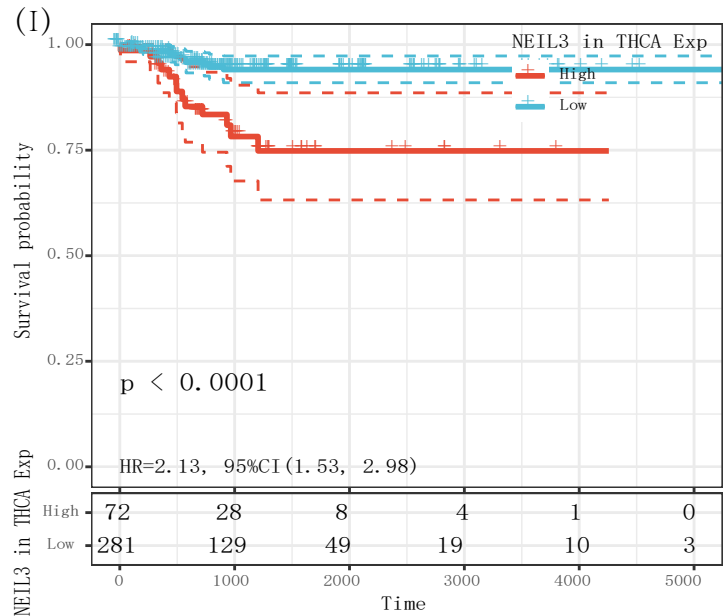

Supplement: Supplementary file 1 [file cancers-15-00109-s001.zip › Supplementary Fig S4 DFI.pdf]

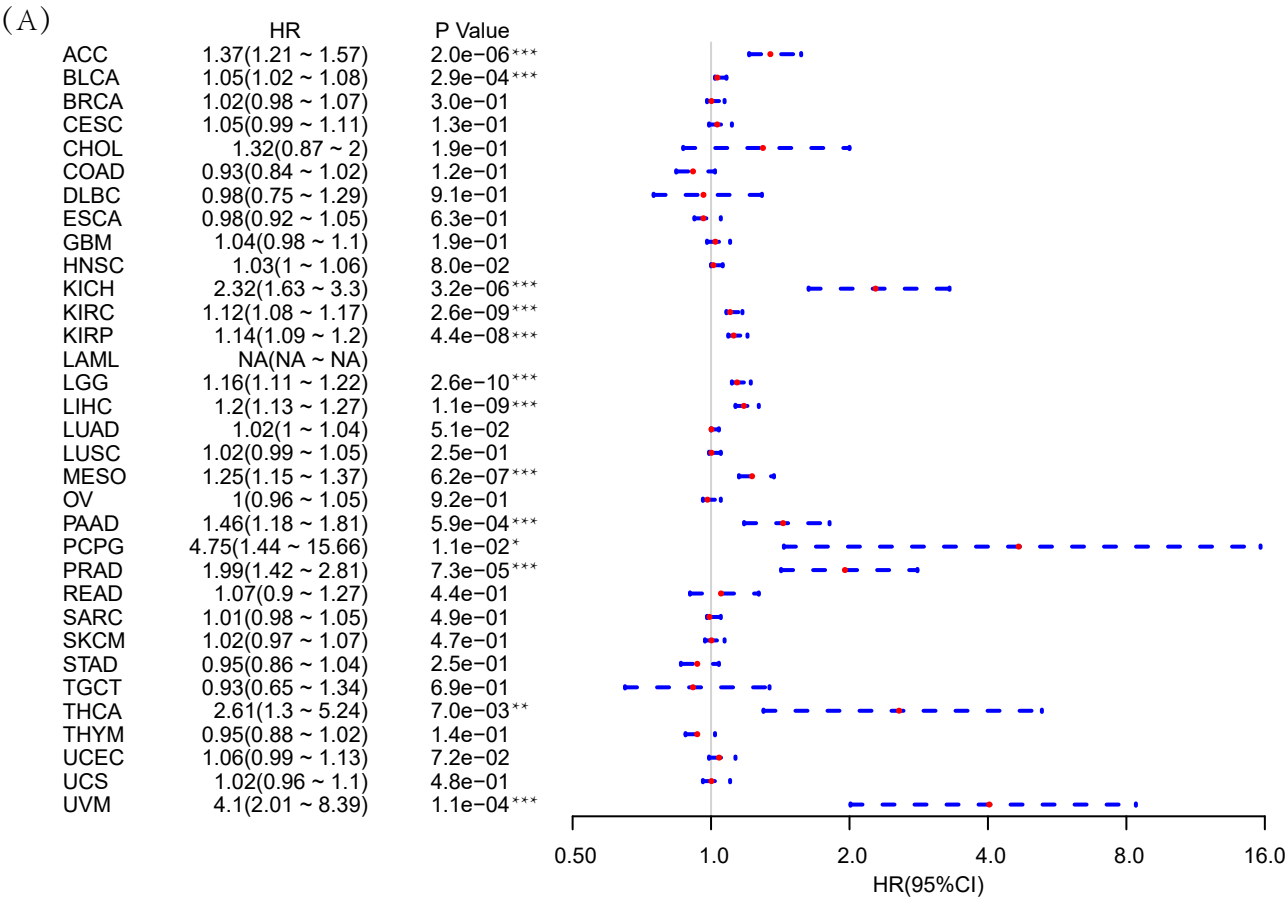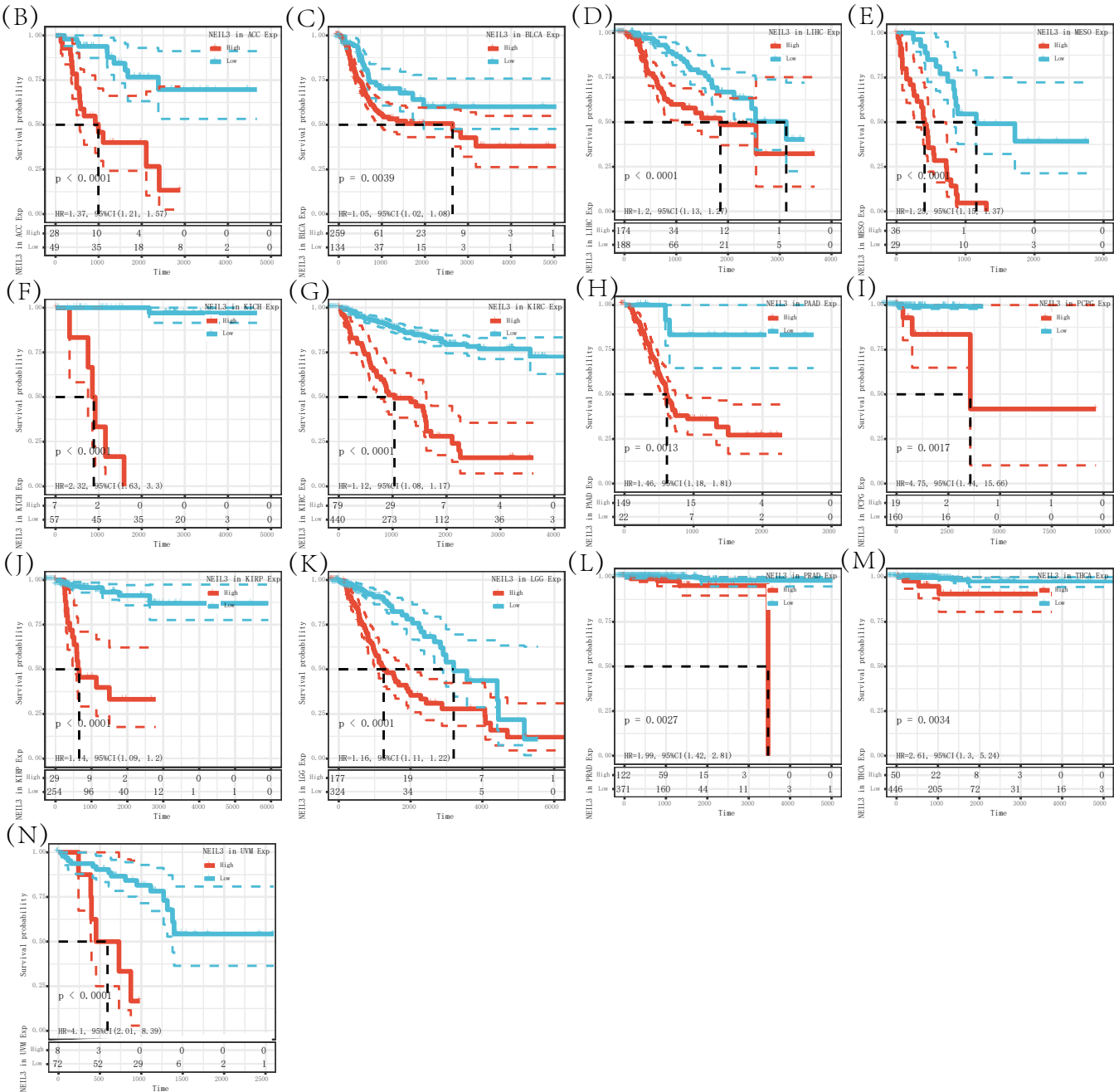

Supplement: Supplementary file 1 [file cancers-15-00109-s001.zip › Supplementary Fig S5 DSS.pdf]

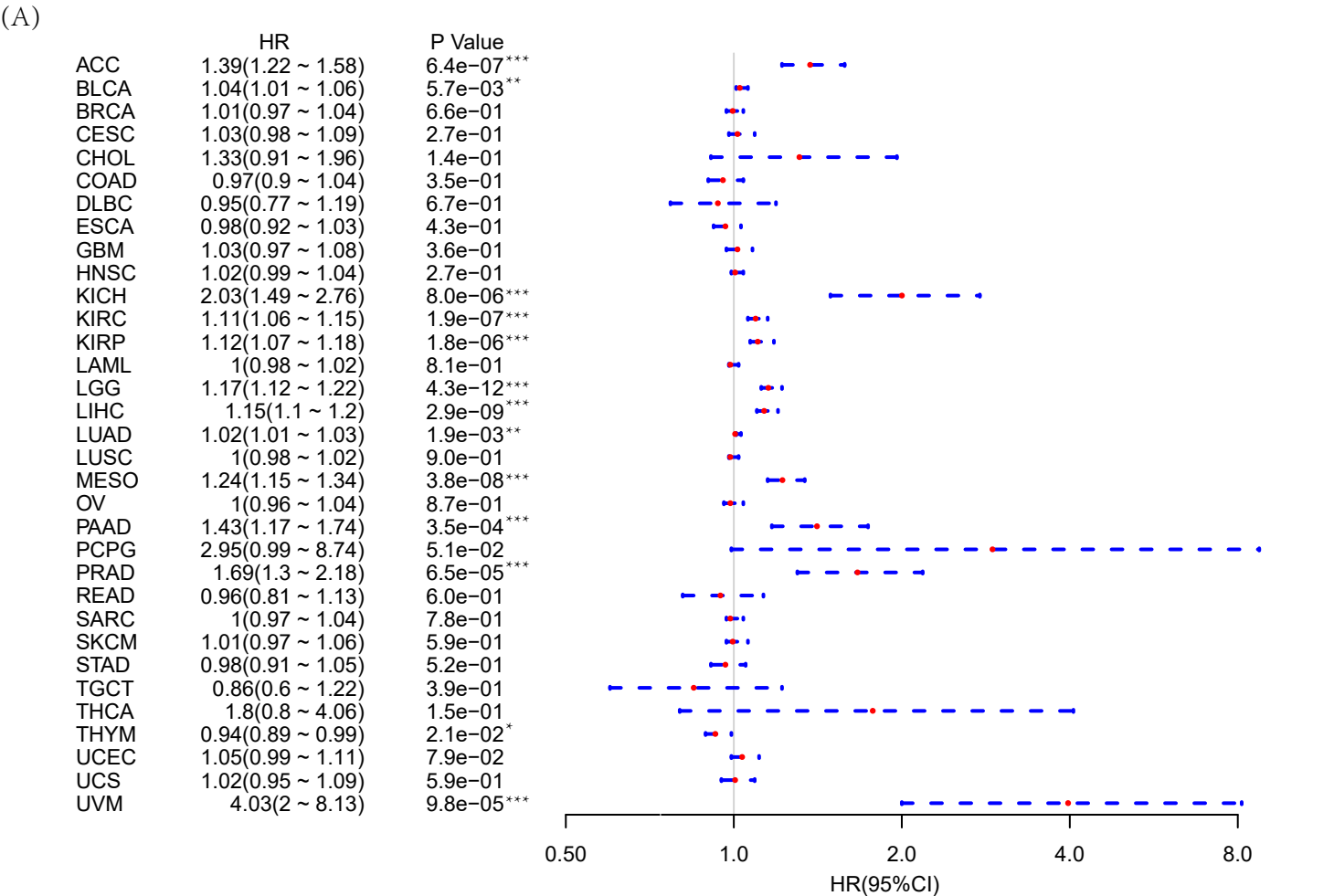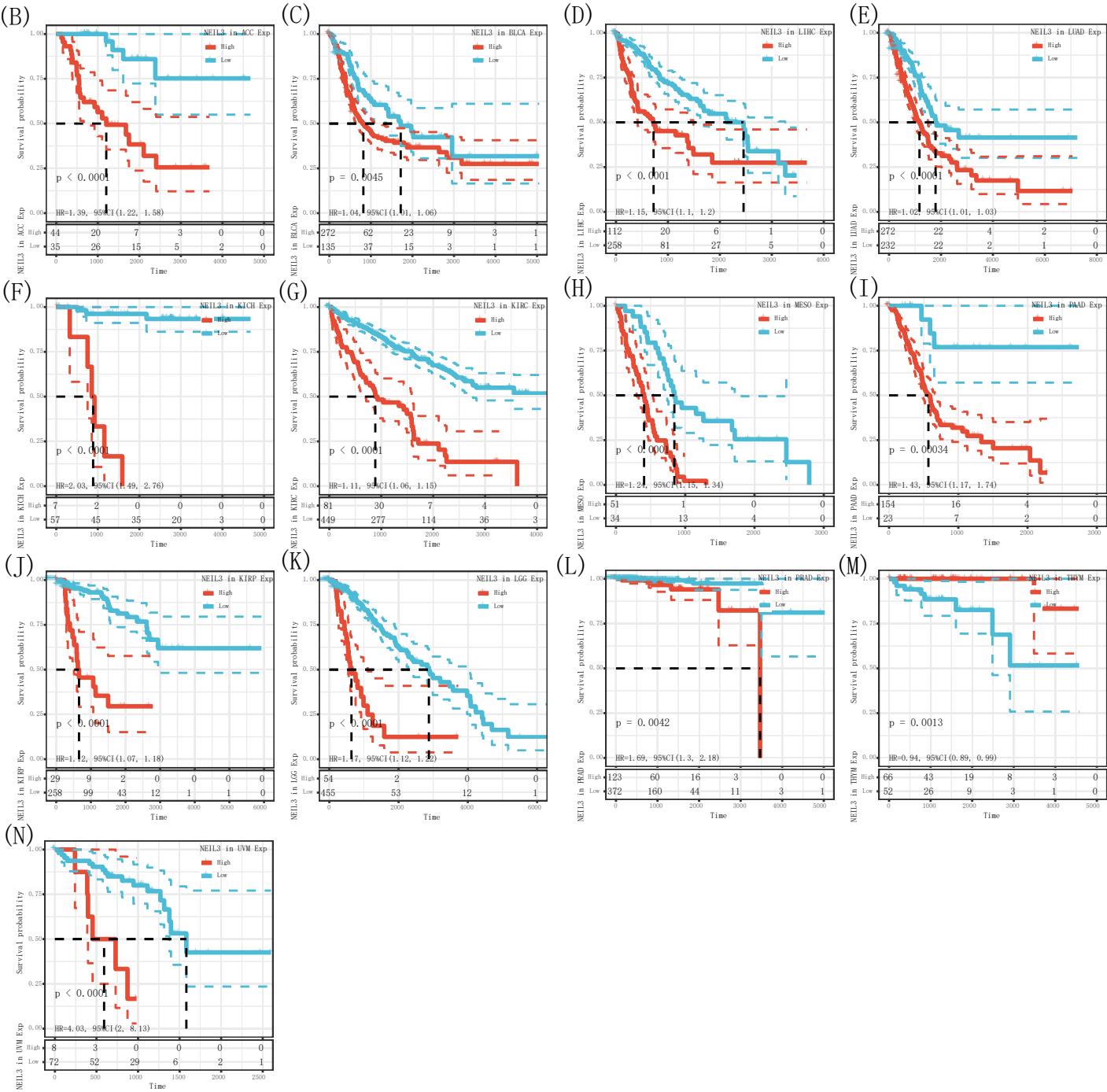

Supplement: Supplementary file 1 [file cancers-15-00109-s001.zip › Supplementary Fig S6 OS.pdf]

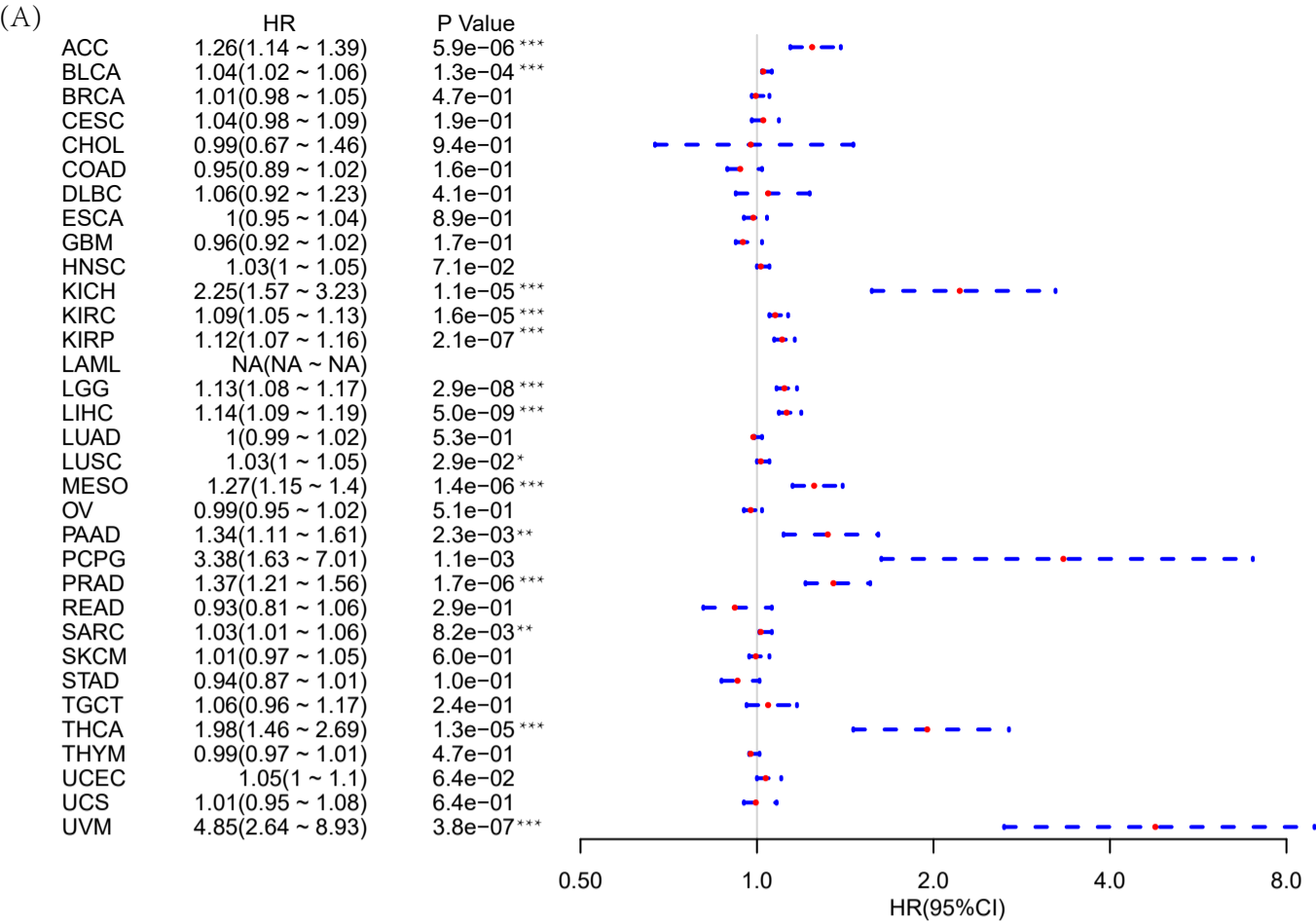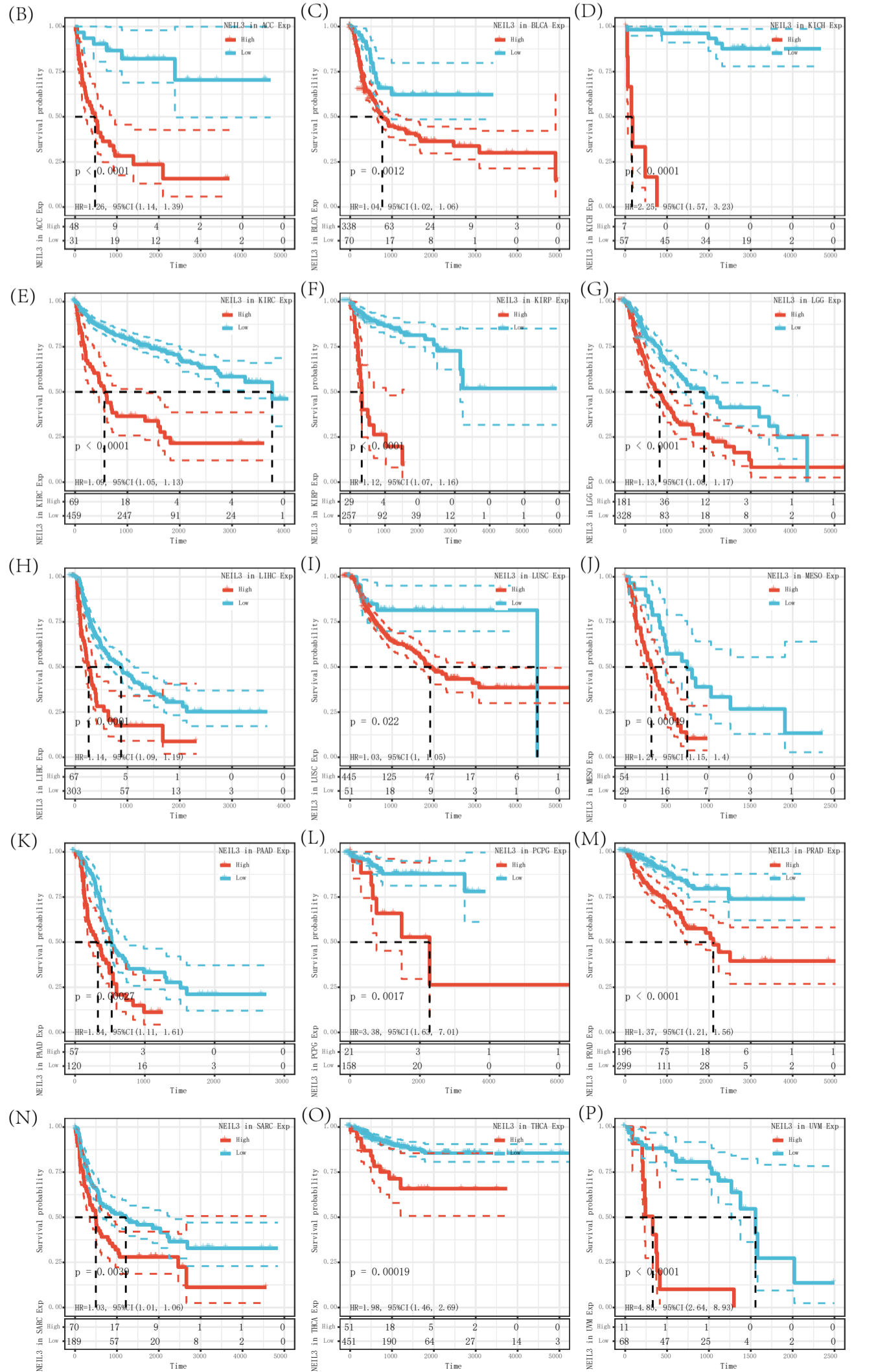

Supplement: Supplementary file 1 [file cancers-15-00109-s001.zip › Supplementary Fig S7 PFI.pdf]

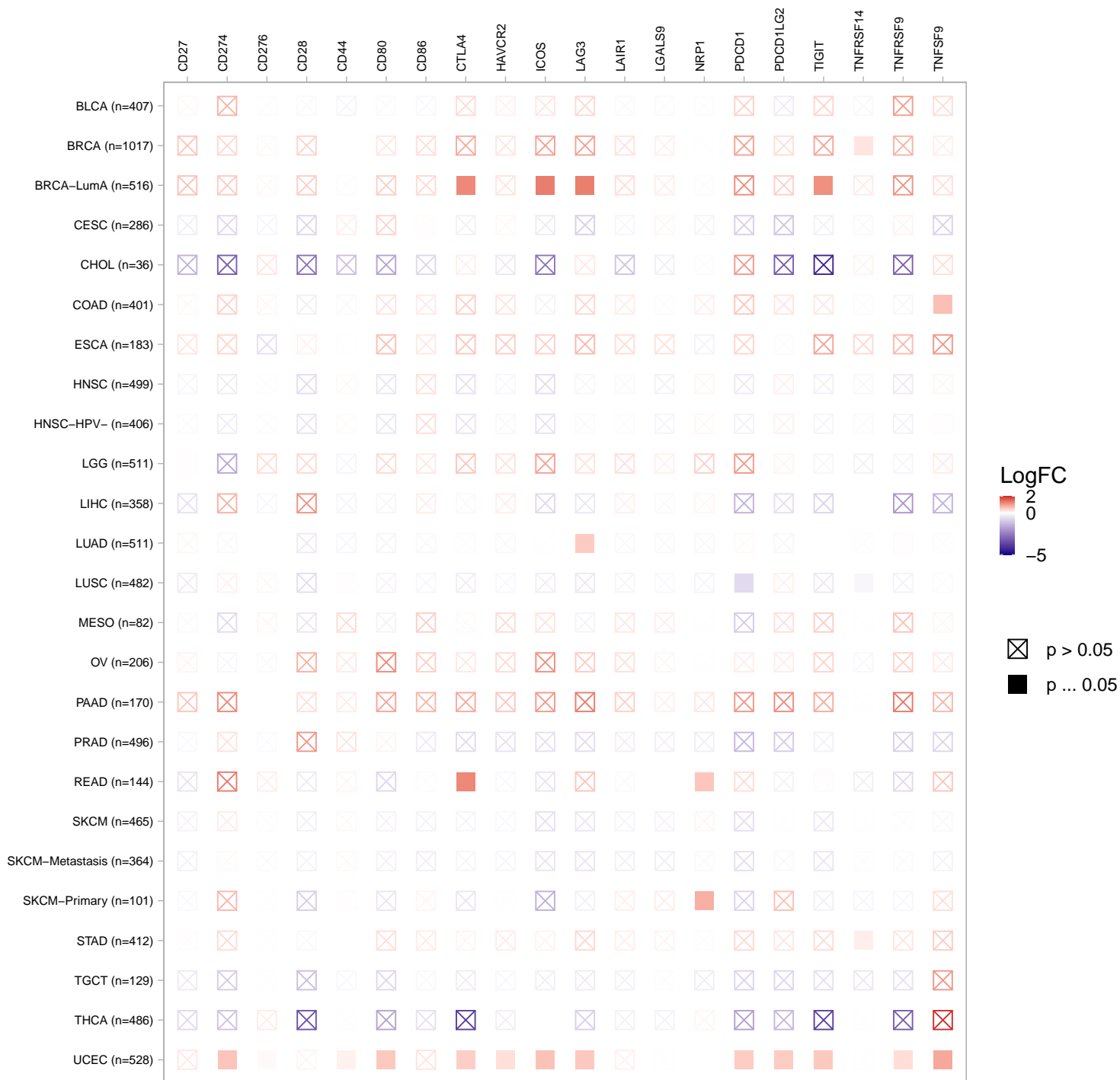

Supplement: Supplementary file 1 [file cancers-15-00109-s001.zip › Supplementary Fig S8 mutation pancancer.pdf]

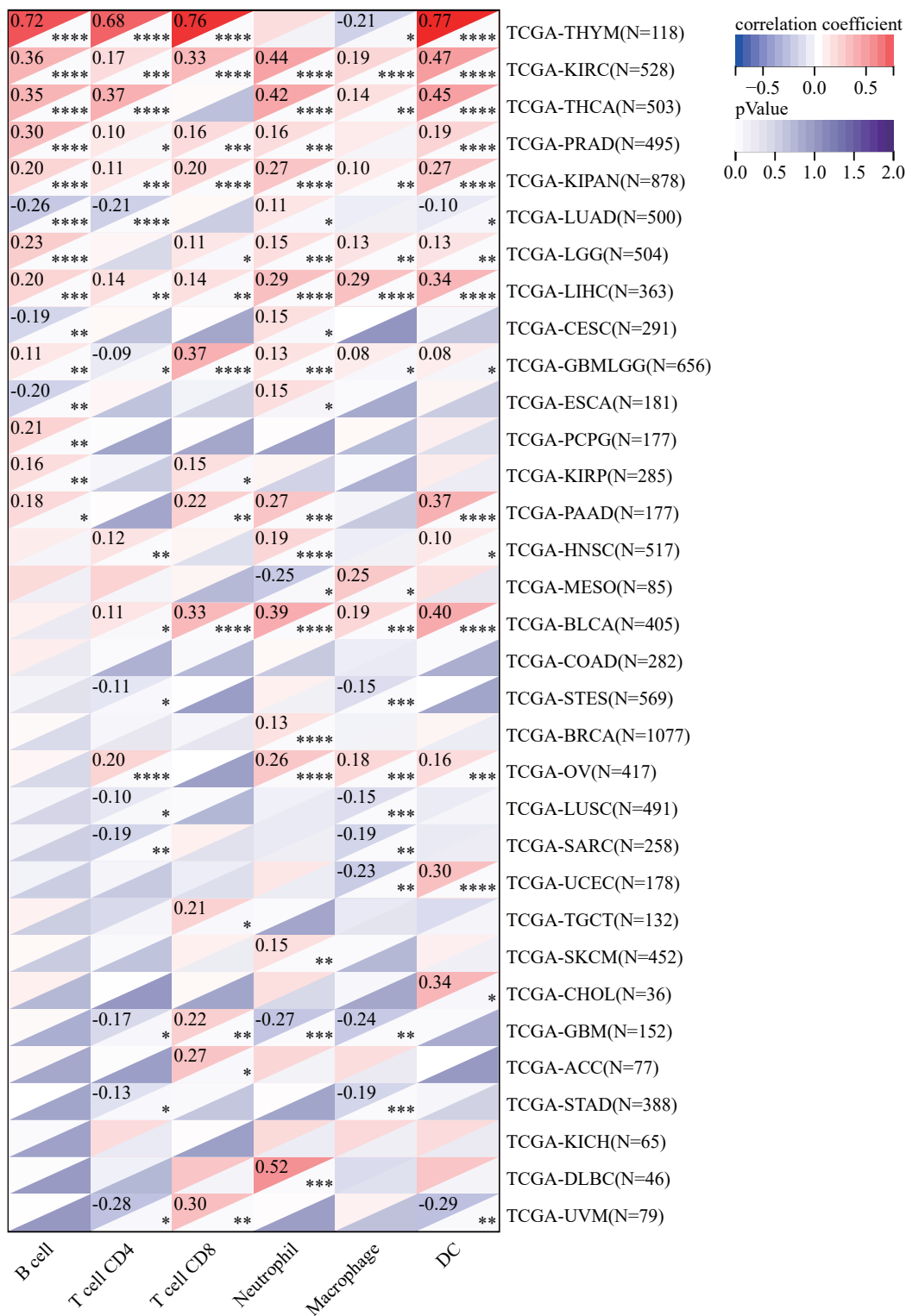

Supplement: Supplementary file 1 [file cancers-15-00109-s001.zip › Supplementary Fig S9 33cancers immune infiltration.pdf]

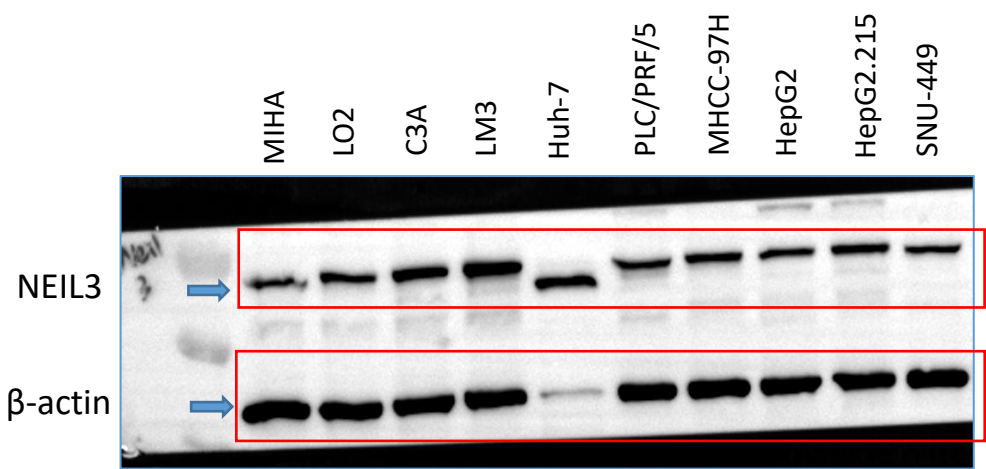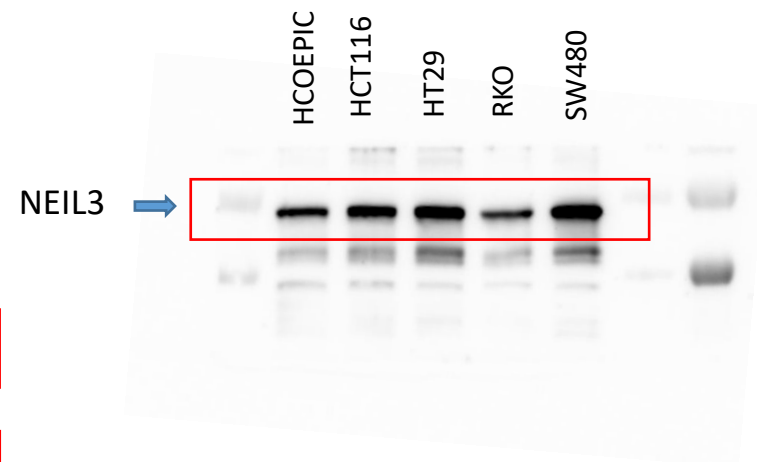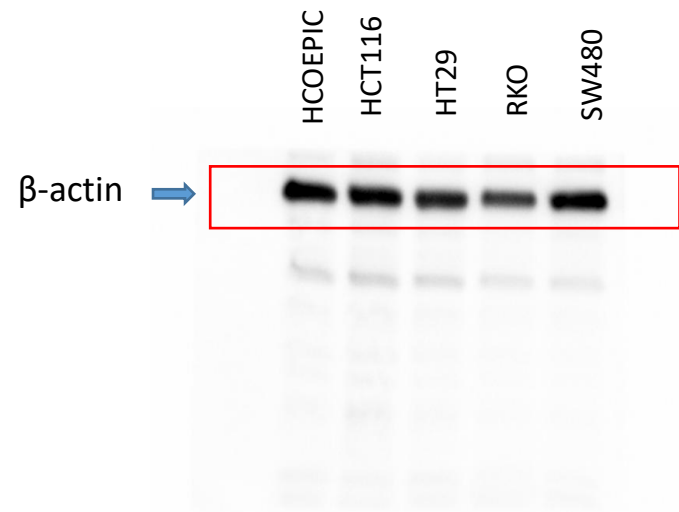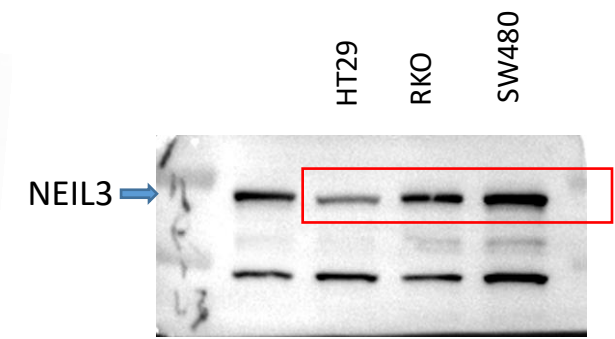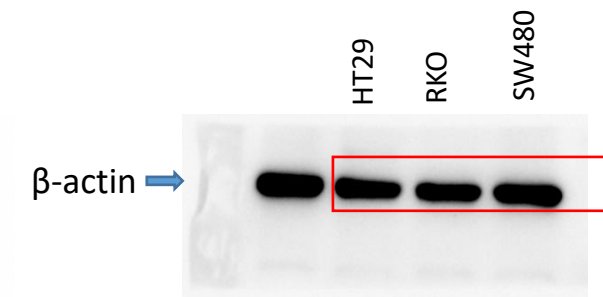

Supplement: Supplementary file 1 [file cancers-15-00109-s001.zip › Supplementary Figure S17 Original Western Blot.pdf]
